# The IFMIF-DONES Project: Design Status and Main Achievements Within the EUROfusion FP8 Work Programme

**DOI:** 10.1007/s10894-022-00337-5

**Published:** 2022-10-26

**Authors:** D. Bernardi, A. Ibarra, F. Arbeiter, F. Arranz, M. Cappelli, P. Cara, J. Castellanos, H. Dzitko, A. García, J. Gutiérrez, W. Królas, F. Martin-Fuertes, G. Micciché, A. Muñoz, F. S. Nitti, T. Pinna, I. Podadera, J. Pons, Y. Qiu, R. Román

**Affiliations:** 1 ENEA, Brasimone, Italy; 2 Consorcio IFMIF-DONES España, Granada, Spain; 3 KIT, Karlsruhe, Germany; 4 CIEMAT, Madrid, Spain; 5 ENEA, Frascati, Italy; 6 F4E, Garching, Germany; 7 INAIA, Universidad de Castilla-La Mancha, Toledo, Spain; 8 Empresarios Agrupados International (EAI), Madrid, Spain; 9 IFJ PAN, Kraków, Poland

**Keywords:** IFMIF-DONES, WPENS, Fusion neutron source, Fusion materials irradiation, DONES-PreP, LIPAc

## Abstract

International Fusion Materials Irradiation Facility-DEMO-Oriented NEutron Source (IFMIF-DONES) is a high-intensity neutron irradiation facility for qualification of fusion reactor materials, which is being designed as part of the European roadmap to fusion-generated electricity. Its main purpose is to study the behavior of materials properties under irradiation in a neutron flux able to simulate the same effects in terms of relevant nuclear responses as those expected in the first wall of the DEMO reactor which is envisaged to follow ITER. It is thus a key facility to support the design, licensing and safe operation of DEMO as well as of the fusion power plants that will be developed afterwards. The start of its construction is foreseen in the next few years. In this contribution, an overview of the IFMIF-DONES neutron source is presented together with a snapshot of the current engineering design status and of the relevant key results achieved within the EUROfusion Work Package Early Neutron Source (WPENS) as part of the 2014–2020 EURATOM Research and Training Programme, complementary to the EU Horizon 2020 Framework Programme (FP8). Moreover, some information on the future developments of the project are given.

## Introduction

A fusion-relevant neutron source is a more than three decades long pending step for the successful development of fusion energy. In DEMO, the demonstrator fusion power plant to be constructed after the ITER machine, the deuterium-tritium nuclear fusion reactions will generate neutron fluxes in the order of 10^18^ m^− 2^ s^− 1^ with an energy of 14.1 MeV that will collide with the reactor chamber first wall. The latter will be the worst exposed component, potentially undergoing a damage dose rate in excess of 15 dpaNRT displacement per atom per full power year (dpa/fpy) of operation [1]. Safe design, construction and licensing of a fusion reactor by the concerned Regulatory Agency will demand the understanding of the materials degradation under the neutrons bombardment during the lifetime of the facility, an issue also affecting protection of investment and operational costs. The plasma facing components shall withstand the severe working conditions to which they are subjected during reactor operation without significant impact on their dimensional stability and mechanical and physical properties [2, 3].

IFMIF-DONES (International Fusion Materials Irradiation Facility-DEMO-Oriented NEutron Source) will be the relevant neutron source of unparalleled power and performance fulfilling the aforementioned needs [4]. It will generate a neutron flux with a broad energy distribution mimicking the effects of the typical neutron spectrum of a (D-T) fusion reactor by means of Li(d,xn) nuclear stripping reactions [5].

The journey to achieve the current maturity of such a high-power neutron source has been long and winding [6]. The seminal proposal towards a fusion-relevant neutron source based on Li(d,xn) nuclear reactions was published in 1975 [7]. The US Fusion Materials Irradiation Test (FMIT) facility [8, 9] produced the first experimental evidence of the feasibility of the concept. After its early termination in 1984 due to economic and technical difficulties, the International Energy Agency (IEA) fostered a series of meetings, at the end of which consensus was attained within the materials scientist community to endorse FMIT´s Li(d,xn) concept [10] through the International Fusion Materials Irradiation Facility (IFMIF) project. The report on the IFMIF conceptual design was issued in 1996 [11] followed by a Comprehensive Design Report [12] in 2004.

The IFMIF/EVEDA project (acronym that stands for IFMIF Engineering Validation and Engineering Design Activities) under the Broader Approach Agreement between Japan and EURATOM was approved in 2007, concurrently with the ITER agreement, with the mandate to produce an integrated engineering design of IFMIF and the data necessary for future decisions on the construction, operation, exploitation and decommissioning of the neutron source, as well as to validate continuous and stable operation of each IFMIF subsystem [13, 14].

The engineering design activities were accomplished on schedule with the release of the IFMIF Intermediate Engineering Design Report (IIEDR) in June 2013 [15].

In recent years, a Fusion Roadmap was developed in Europe [16] which is based on the objective of electricity production from fusion reactor around mid of the century, speeding up the design and construction phase of DEMO (foreseen to be started around 2040) and, at the same time, reducing the neutron dose requirements on the materials. Thus, an initial DEMO phase is foreseen with a maximum dose around 20 dpa for components integration testing and a second DEMO phase with a maximum dose around 50 dpa for performance testing of more advanced technological solutions [1].

In this way, the requirements for the early phase of the neutron source are significantly reduced, opening the possibility of a staged approach to IFMIF in which its construction can be developed in phases, the first one being focused on the early DEMO needs, hence giving rise to the IFMIF-DONES project [17]. This staged approach will allow a broader distribution of the required investment along the time as well as some relaxed specifications for the neutron source design.

## The IFMIF-DONES Facility

Following the aforementioned approach, a specific action was initiated in 2015 in the framework of the Work Package Early Neutron Source (WPENS) of the EUROfusion Consortium, as part of the 2014–2020 EURATOM Research and Training Programme. This programme was funded as a complementary to the EU Horizon 2020 Framework Program (FP8) and it has been recently extended within the Horizon Europe (FP9) programme for the years 2021–2025. The WPENS objective is to develop the design of IFMIF-DONES up to a level that allows to be ready for the start of its construction phase.

In the following, a brief illustration of the mission, of the top-level requirements and of the basic plant configuration of the facility is given.

### Mission and Top-Level Requirements

The mission of IFMIF-DONES is to provide a neutron source producing high energy neutrons at sufficient intensity and irradiation volume in order to:

1. Generate materials irradiation test data for the design, licensing, construction and safe operation of the fusion demonstration power reactor (DEMO), with its main characteristics as defined by the EU Roadmap [16] under simulated fusion environment relevant to anticipated needs in radiation resistance for the structural materials in DEMO [18, 19].
2. Generate a data base for benchmarking of radiation responses of materials hand in hand with computational material science.

Moreover, the material properties dataset generated by IFMIF-DONES will potentially be used also for comparison with larger volumes irradiations data obtained from other non-fusion installations (like fission reactors, spallation sources, etc…).

Additionally, given the fact that IFMIF-DONES will be available during ITER operation, the possibility that IFMIF-DONES could assist ITER in some aspects of its nuclear operation phase should not be disregarded.

The mission of IFMIF-DONES is translated into a number of high-level technical requirements as shown in Table 1.

**Table 1:** IFMIF-DONES top-level requirements

Requirement	Description/value	Remarks
Neutron spectrum	Able to simulate relevant nuclear responses (primary recoil spectrum, transmutations, gas production) of early DEMO first wall	
Accumulated damage versus irradiation volume	20–30 dpa_NRT_ (Fe) in < 2.5 years over 300 cm^3^ 50 dpa_NRT_ (Fe) in < 3 years over 100 cm^3^	> 8–12 dpa/fpy in 0.3 l > 16 dpa/fpy in 0.1 l
Irradiation temperature	250–550 °C	Actively controlled
DPA gradient	< 10%	Over a gauge volume corresponding to miniaturized specimens
Temperature gradient	< 3%	Same volume as for DPA gradient. Long-term stability in the same order
Design lifetime	> 30 years	> 20 y operation lifetime
PIE	External laboratories	See Ref. [20]

IFMIF-DONES shall be designed for a minimum lifetime of 30 years, with at least 20 years of irradiation experiments on a three-shift basis 24/7. Additionally, an average operational availability goal of 70% over calendar year (or 75% over scheduled operation time) has been established as a target for normal operation phase.

As far as the Post Irradiation Examination (PIE) is concerned, a list of potential external laboratories presently existing in Europe with the capabilities to analyze and characterize the irradiated samples coming from IFMIF-DONES has been preliminarily identified in the framework of the DONES-PreP Project (see “Project Evolution and Achieved Milestones” section). This list, together with a brief description of the identified facilities, is reported in [20].

### Plant Configuration

IFMIF-DONES is configured as an accelerator-driven facility generating a 125 mA continuous-wave (CW) deuteron beam that, accelerated up to 40 MeV and shaped to have a nominal rectangular footprint, impinges on a 25 mm thick liquid lithium screen cross-flowing at about 15 m/s in front of it. The nuclear stripping reactions between D+ and Li generate a large number of neutrons that interact with the material samples housed in the High Flux Test Module (HFTM) located immediately behind the lithium target.

The main features of the IFMIF-DONES facility and their major differences with respect to the IFMIF configuration are summarized in Table 2.

**Table 2:** IFMIF-DONES versus IFMIF specifications

	IFMIF	IFMIF-DONES
N. of accelerators	2	1
Beam current	250 mA	125 mA
Beam energy	40 MeV	40 MeV
Beam power	10 MW	5 MW
Beam footprint size	(20 × 5) cm	from (10 × 5) cm to (20 × 5) cm
Test modules	HFTM, MFTM, LFTM	HFTM only
PIE	On-site	Off-site

In particular, the IFMIF-DONES lithium target shall have the same physical dimensions of the IFMIF one. On the other hand, differently from IFMIF where three irradiation modules were foreseen, i.e. the High Flux Test Module (HFTM), the Medium Flux Test Module (MFTM) and the Low Flux Test Module (LFTM), the reference approach in IFMIF-DONES is that only the HFTM will be present, with an enlarged thickness compared to the IIEDR configuration [15] to gain irradiation volume. This is justified by the fact that IFMIF-DONES will be aimed at providing only high flux irradiation of structural materials. Nevertheless, other irradiation modules or experiments might be included as well, as a result of additional needs to be identified in the future.

Another difference with respect to IFMIF is that IFMIF-DONES will require the transport of the activated samples for the Post Irradiation Examination (PIE) that will be carried out in external laboratories.

Furthermore, the possible future upgrade to the full IFMIF is considered in the design of the facility.

**Fig. 1:**
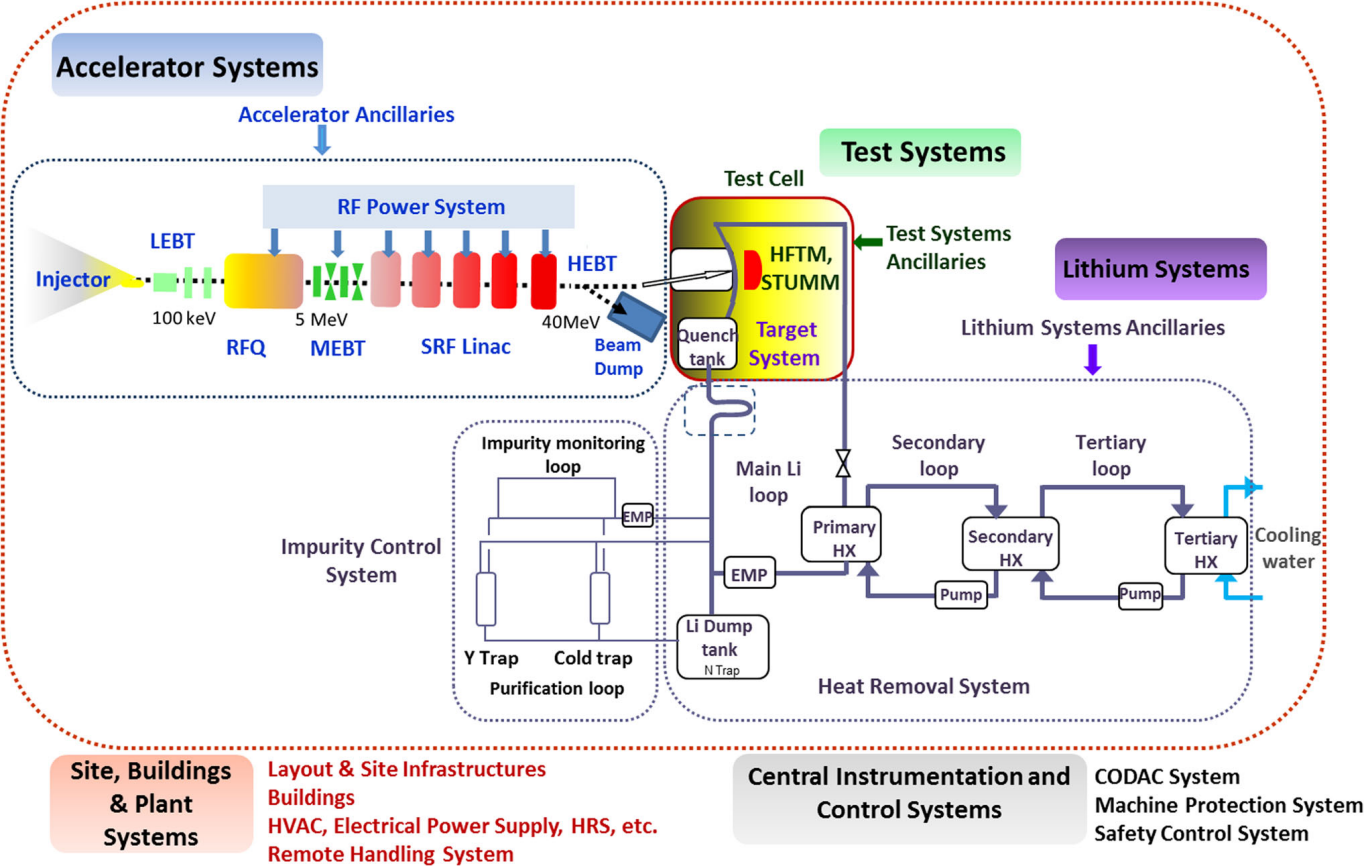
IFMIF-DONES plant configuration

Figure 1 shows a schematic view of the current configuration of the IFMIF-DONES plant.

The IFMIF-DONES Plant Breakdown Structure (PBS) identifies five major areas or groups of systems (PBS level 1): the Site, Building and Plant Systems (PBS 3.0); the Test Systems (PBS 4.0); the Lithium Systems (PBS 5.0); the Accelerator Systems (PBS 6.0); and the Central Instrumentation and Control Systems (PBS 8.0).

## Overview and Status of IFMIF-DONES Design

In this Section, a brief description of each of the abovementioned PBS areas is provided, with a focus on the most relevant engineering challenges and on the status of the concerned systems design.

### Accelerator Systems

The IFMIF-DONES accelerator [21] has the function to generate, accelerate and transport the D+ beam with the required characteristics up to the lithium target. Its design is based on the IFMIF one, although some important differences are present. The incident angle to the target has been kept at 9° (as in IFMIF) to account for a future upgrade of the facility with a second accelerator.

**Fig. 2:**
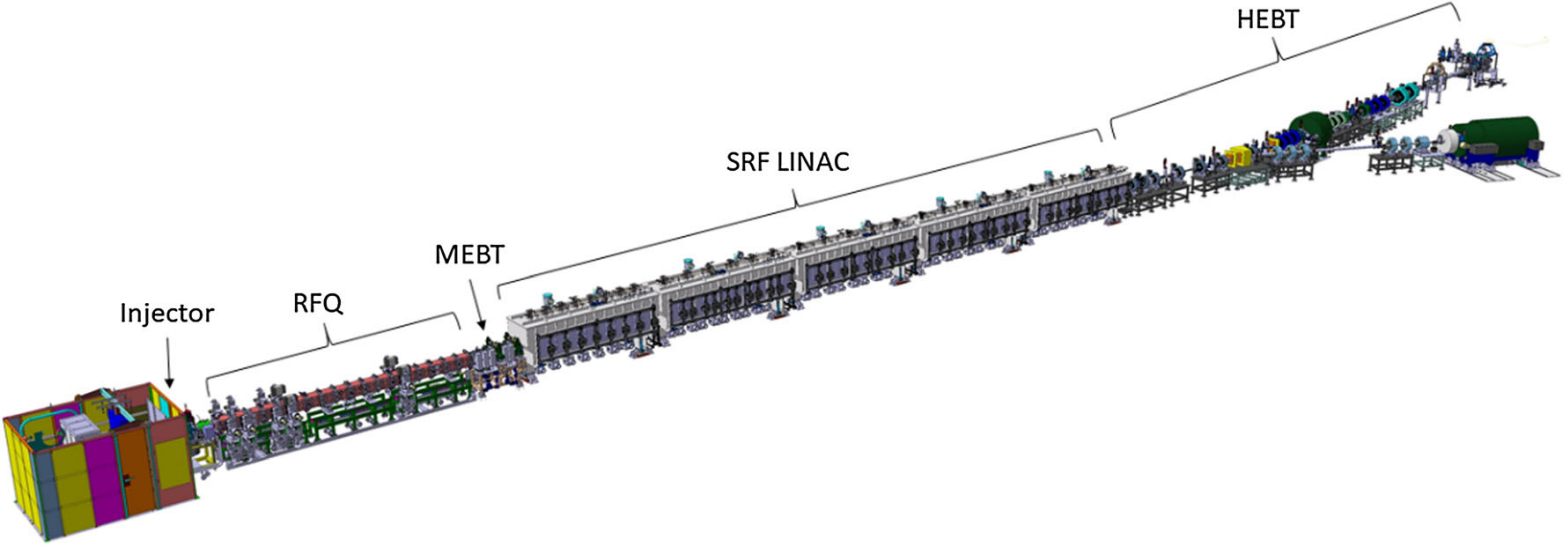
Schematic of the IFMIF-DONES accelerator

A schematic of the whole system configuration is depicted in Fig. 2. Three main parts can be identified: the ion source, the accelerating stage and the final high energy transport section.

**Fig. 3:**
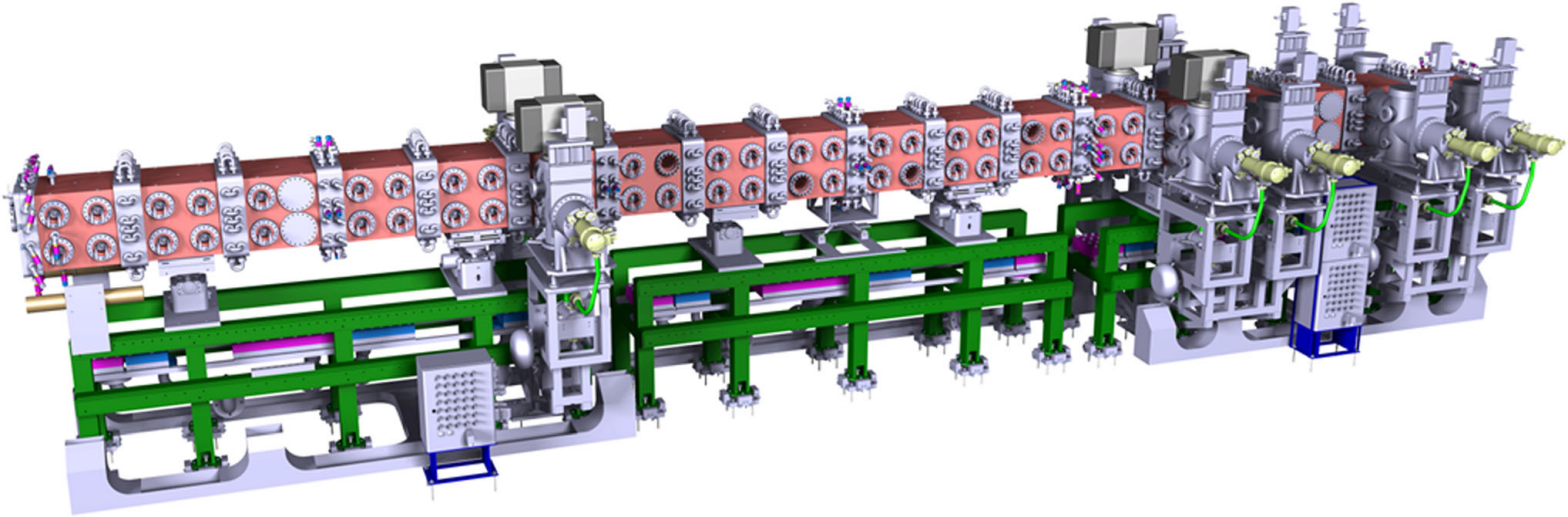
IFMIF-DONES radiofrequency quadrupole (RFQ)

The D+ beam is produced by an Electron Cyclotron Resonance ion source [22] extracted and accelerated in continuous wave (CW) mode up to 100 keV. A Low Energy Beam Transport (LEBT) section guides the deuteron beam from the source to a Radio Frequency Quadrupole (RFQ) (Fig. 3). The RFQ [23] is designed to bunch and accelerate the beam from 100 keV up to 5 MeV. The total length of the IFMIF-DONES RFQ is around 10 m, which makes it the longest in the world.

Due to these unprecedented features, the RFQ design represents a big challenge which could potentially become a severe risk for the successful development of the whole facility and thus it needs to be properly validated. For this reason, the full-scale prototype LIPAc, (see “Validation Results and Lessons Learned” section) has been constructed and is now under commissioning in Japan with the main goal to demonstrate the possibility to reliably operate the accelerator frontend components under the real IFMIF-DONES conditions. Indeed, the performance of the RFQ has been successfully demonstrated in 2019 at least for low duty cycle (0.1%) regime, as described in the following (see “LIPAc Commissioning Tests” section). Next step in LIPAc will be to qualify the RFQ for CW operation. Potential risk of misalignments of the RFQ components might occur during the life of the facility. Therefore, suitable registration devices are envisaged to be installed on the RFQ modules supports, which are capable of adjusting the modules position (besides ensuring their initial alignment during installation) in case some misalignments are detected. The effectiveness of such devices has been proved in LIPAc where an alignment precision below 50 μm was obtained for the beam axis of both ends [24]. Another major problem linked with the RFQ operation is the strong erosion that is expected on the inner vanes of the first modules, which has impact on the resonant frequency as well as on the RF field distribution and beam losses. To counteract this phenomenon, some mitigating actions (e.g., varying the inlet cooling water temperature to retune the resonance frequency and locally restore the field perturbation) can be undertaken to increase the lifetime of the RFQ to some extent (a couple of years extension is the current rough estimation). Over longer periods, the replacement of the whole modules or of some parts of them could be needed. The outcomes of LIPAc operation in CW will provide further information related to the erosion issue. Furthermore, some alternative countermeasures are also currently being considered at design level in order to avoid or significantly reduce the insurgence of the problem and thus increase the availability of the system.

**Fig. 4:**
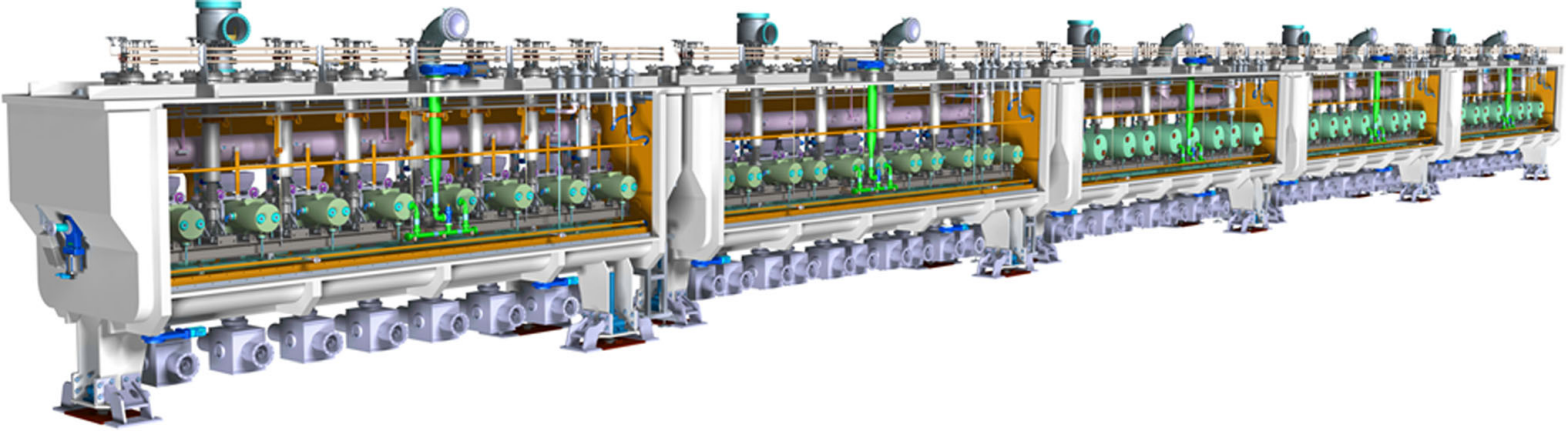
IFMIF-DONES superconducting RF Linac

The beam exiting the RFQ is injected into a Medium Energy Beam Transport (MEBT) section [25] which provides the matching interface between the RFQ and the Superconducting Radio Frequency Linac (SRF Linac). This is done by removing out-of-acceptance particles with scrapers, transporting the beam in the transverse plane with quadrupole magnets, and modifying the longitudinal space to the SRF Linac input requirements with re-buncher cavities. The beam is then accelerated by the SRF Linac [26] through a set of superconducting cavities and focusing solenoids. The superconducting RF Linac consists of five cryomodules cooled by liquid helium and separated by warm sections (Fig. 4). The acceleration is given by superconducting Half Wave Resonators made of niobium and with a shape optimized for two different deuteron velocities, the first 19 ones for the first two cryomodules, and the other 27 for the last three. At the exit of the last cryomodule the beam reaches the expected power of 5 MW (125 mA @ 40 MeV).

All the accelerating Radio Frequency cavities are powered by a Radio-Frequency (RF) power system based on 175 MHz high power solid state RF stations with a CW output power level up to 200 kW [27]. Additionally, the MEBT is powered by two smaller solid-state amplifiers rated at 16 kW CW. A total of 56 RF stations are needed (8 for the RFQ, 2 for the MEBT and 46 for the SRF Linac).

Downstream of the Linac, a High Energy Beam Transport (HEBT) line [28] guides the deuteron beam towards the target and shapes it by the use of magnetic elements to obtain a rectangular footprint that impinges on the lithium jet. The HEBT is designed flexible enough to deliver a beam with (horizontal × vertical) footprint dimensions ranging from (10 × 5) cm to (20 × 5) cm.

In addition, the Accelerator Systems Ancillaries provide all the necessary equipment and utilities for the required supporting services (primary cooling, electrical power, vacuum system, gas distribution, liquid He supply, etc.).

The IFMIF-DONES accelerator will be one of the most powerful accelerators in the world and, among those, the one with the highest average deuteron current ever. This latter feature, in particular, poses severe issues due to the associated high space charge effect which tends to defocus the beam, especially in the low/medium energy section, and thus must be carefully addressed.

Additionally, besides the already mentioned RFQ’s long length and the flexibility required to the HEBT section to provide different beam footprints, the accelerator systems will have to operate in CW mode with a high availability requirement (87%). This is needed in order to achieve the high-level requirement for the global inherent availability (i.e., referred to the scheduled operation time) of the whole facility which is set at 75% to maximize the irradiation time while ensuring a suitable shutdown period (20 days/year) for preventive maintenance. The target of 87% availability for the AS comes from the reasonable allocation of the said 75% global availability among the different systems of the Plant (Accelerator Systems: 87%; Lithium Systems: 94%; Test Systems: 96%; Plant Systems: 98%; Central Instrumentation and Control Systems: 98%).

All of the unique and demanding features mentioned above make the IFMIF-DONES accelerator a very challenging system.

At the end of the FP8 activities, various systems of the IFMIF-DONES accelerator have reached a high level of design maturity, like the injector, the RFQ and the MEBT as the validation tests of their prototypes in LIPAc have advanced [29, 30] as well as the commissioning of similar accelerators around the world (see “LIPAc Commissioning Tests” section). As a main conclusion, it is possible to state that the design of the accelerator systems seems feasible and no showstoppers are identified so far.

### Lithium Systems

The Lithium Systems (LS) have the function to provide the liquid lithium jet which interacts with the D+ beam and to evacuate the heat power deposited in the lithium by the high-energy deuterons. They comprise three main systems: the Target System; the Heat Removal System; and the Impurity Control System.

**Fig. 5:**
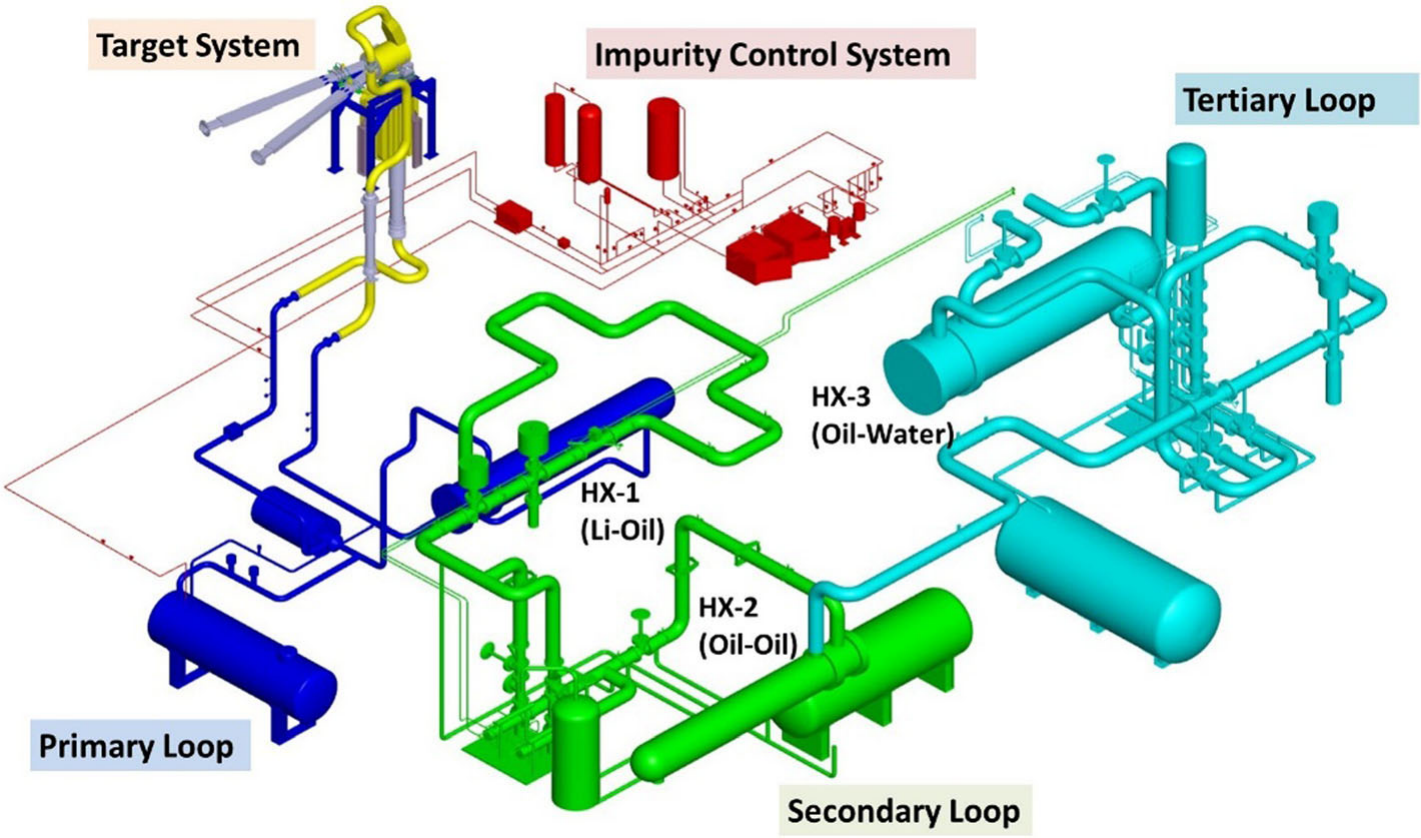
The IFMIF-DONES lithium systems

A sketch of these systems is shown in Fig. 5.

#### The Target System

The Target System (TSY) includes the components located inside the Test Cell (see “The Test Cell” section) as well as those penetrating its concrete shielding walls, namely the inlet an outlet plug assemblies and the two beam ducts connected to the accelerator line and to the lithium jet diagnostic module, respectively. The latter is equipped in particular with a laser-based distance meter for the measurement of the lithium jet thickness. The main component of the TSY is the Target Assembly (TAA) which includes the Backplate (BP) with the concave-shaped open channel exposed to the accelerator vacuum (10^− 2^ Pa). The TAA provides a free-surface lithium jet flowing on the BP at high speed (15 m/s) in front of the D+ beam, which is able to sustain the 5 MW power deposited in it while avoiding boiling and significant evaporation of the lithium. The concavely curved channel is needed to create an inner pressure inside the lithium and thus locally raise its boiling temperature. The TAA is also required to stably maintain the proper thickness (25 mm) of the lithium jet in order to guarantee the delivery of a constant neutron flux to the materials samples placed behind it and to fully stop the D+ beam within the lithium layer so avoiding its impingement on the Backplate (which would lead to the damage of the component in a very short time). The TSY is designed to manage a thermal flux that can reach up to 1 GW/m^2^ (in the 10 × 5 cm^2^ footprint configuration) with a lithium temperature increase of less than 50 °C through the target and a design lithium inlet temperature of 300 °C.

**Fig. 6:**
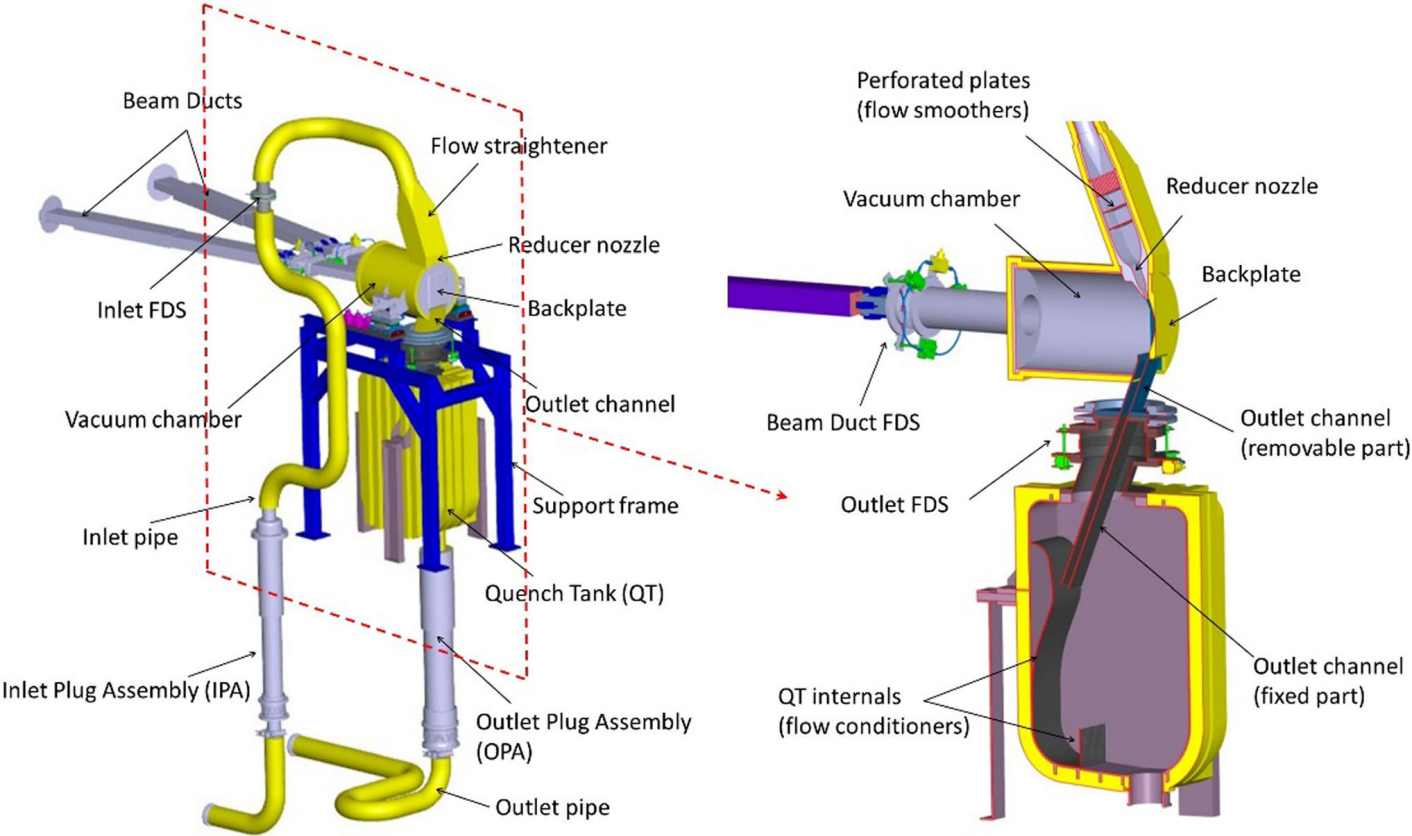
The IFMIF-DONES target system

A sketch of the whole TSY is shown in Fig. 6.

The BP is the most exposed component to the neutron flux (even more than the materials samples) and thus it needs to be periodically replaced. In IFMIF-DONES, the TSY adopts an integral concept where the BP is welded to the removable part of the TAA. The latter is attached to the fixed part of the structure by means of flanged connections (at inlet and outlet pipes and at Beam Ducts interfaces) using a Fast Disconnecting System (FDS) which allows easy and quick connection/disconnection operations by Remote Handling tools. This concept implies that the BP is changed together with the removable part of the TAA every approximately 11 months during the yearly preventive maintenance of the facility.

#### The Heat Removal System

The Heat Removal System (HRS) mainly comprises the primary loop (Li loop) with its Dump Tank and the electromagnetic pump which circulates the lithium between the Target Assembly and the primary lithium-to-oil heat exchanger. Secondary and tertiary oil loops with oil-to-oil and oil-to-water heat exchangers, respectively, transfer the heat from the Li loop to the plant general water cooling system. The HRS is designed to evacuate the heat deposited in the target and to control and maintain a constant lithium temperature at the TAA inlet, irrespective of the beam power.

#### The Impurity Control System

The Impurity Control System (ICS) is a branch line of the HRS which extracts a fraction of the lithium from the main loop and re-injects it after purification and impurities analysis. In particular, the Be-7 (around 150 mg, equilibrium value after one year of irradiation) generated as a by-product of the D-Li interactions must be carefully controlled as it is strongly radioactive and thus plays a major role in the safe operation and maintenance of the Li loop. A moderate amount of tritium (around 4 g/y) is also generated in the target and needs to be strictly managed as well. Other radioactive products are produced by activation of the elements coming from erosion/corrosion of the steel parts of the system. In addition, the nitrogen concentration in the lithium must be accurately controlled as it enhances corrosion and influences the deposition of Be-7 along the loop. It is clear therefore that the ICS is a quite complex system whose design requires addressing many different and competing aspects.

To perform the above functions, the purification branch contains a number of cold traps (to remove elements with temperature-sensitive solubility in lithium, including Be-7) and hydrogen traps (to chemically remove hydrogen isotopes including tritium, in particular, by means of a Y-based getter). In addition to these traps, a nitrogen (hot) trap is required to remove nitrogen by chemical reaction with a nitrogen binding material. The nitrogen trap was included in the ICS in the previous phases of the design but currently a new solution has been developed which foresees the nitrogen trap to be included in the Dump Tank of the main Li loop as an off-line trap. At present, the ICS is undergoing a general reassessment and redesign to ensure that the radioactive impurities produced in the lithium are properly confined in the traps located in dedicated areas of the building. All the radioactive waste generated in the LS (as well as in the rest of the facility) is managed inside the facility by the Solid-, Liquid- and Gas-Radioactive Waste Treatment Systems (S-RWTS, L-RWTS, G-RWTS) which are part of the Plant Systems (see “Main Building and Plant Systems” section). Radioactive waste will be collected, segregated, measured and packaged/conditioned by the RWTS and then taken over by the Spanish National Agency for the nuclear waste management (ENRESA) for their temporary or final storage or disposal in its repository. Activation products like Be-7 and Tritium are mainly captured in the cold and H-traps, respectively, which will be temporarily stored inside the Plant before their immobilization and final disposal. Gaseous effluents are estimated to comply with stringent dose objectives after G-RWTS stages.

Finally, the gas and vacuum subsystems, the heating subsystem, the electric power supply and the lithium and oil recovery subsystems make up the so-called Lithium Systems Ancillaries which support the operation of the LS.

Alike the accelerator, the IFMIF-DONES lithium loop will be a world record loop, as it will become the largest Li circuit in the world containing around 8 m^3^ (about 4 tons) of lithium flowing at nearly 100 l/s.

The design of the LS within the FP8 framework took advantage of the engineering and validation results obtained during the previous IFMIF/EVEDA phase. The main achievements concerned in particular the progress in the Secondary Heat Removal System [31] and in the TSY design [32] which are now quite advanced, although some activities including relevant validation tests for the TSY still need to be finalized in the next phase of the project. A preliminary design of the Li loop and of the ICS was developed but a fundamental revision is currently being carried out to implement new operating conditions of the loop for optimizing the Be-7 management and some relevant changes in the rooms layout to deal with RH and safety requirements resulting from a new traps arrangement.

### Test Systems

**Fig. 7:**
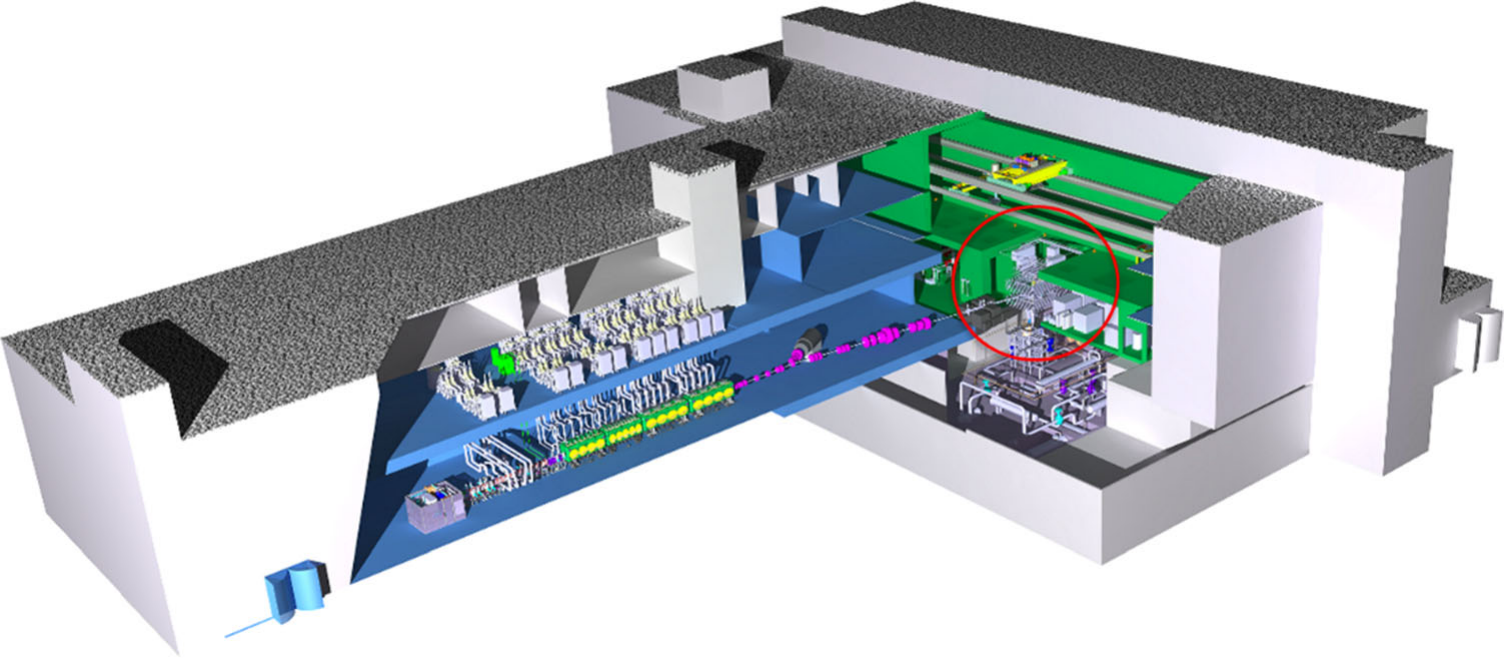
IFMIF-DONES main building showing the TC location (in red circle) (Color figure online)

The Test Systems (TS) are placed at the core of the IFMIF-DONES plant (Fig. 7) and include the Test Cell (TC), the High Flux Test Module (HFTM) housing the specimens to be irradiated and the Start-up Monitoring Module (STUMM) equipped with a wide set of instrumentation to be used during the commissioning phase of the facility. In addition, a set of ancillary systems provides all the necessary services to the TS, such as the electrical power distribution, the cooling media, the vacuum system and the supply and purification of the gases that form the TC atmosphere.

Facilities for Complementary Experiments are also planned to allow for the installation of complementary physics experiments, independent of fusion materials irradiation. Possible areas of these experiments can be nuclear physics, radioisotope production and medicine. These experiments will be using the available neutron flux behind the HFTM and/or a fraction of the deuteron beam deflected at 40 MeV energy.

#### The Test Cell

The Test Cell (TC) is a closed cavity with an opening at the top which physically houses the HFTM (or, alternatively, the STUMM) and some components of the LS, namely the Target Assembly, the Quench Tank (QT) and the lithium pipes interfacing with the Li loop. Below the TC floor, a Test Cell-Lithium Systems Interface Cell (TLIC) is arranged to accommodate the bellows used for the thermal compensation of the inlet and outlet lithium pipes as bellows cannot be credited as confinement barrier.

At the top, two concrete shielding plugs are designed to close the TC during the irradiation period for protecting the Access Cell (AC) located above the TC from radiation.

A Test Cell Cover Plate (TCCP) and a rubber-based sealing gasket are applied to tighten the TC so that a controlled atmosphere inside of it can be achieved. The sealing of the TC is not expected to be exposed to critical radiation levels during operation since it is shielded by the two large concrete slabs (Lower and Upper Shielding Plugs) placed at the top of the TC. Therefore, a rubber-based material is considered safe enough to provide cell tightness.

The complete inner surface of the TC wall is covered by a closed stainless steel liner to maintain the gas tightness and to protect the concrete shielding walls from contact with lithium in case of lithium spill. The atmosphere enclosed by the liner is subject to complex internal flows that has been studied by CFD analysis [33].

Removable piping and cabling plugs (PCPs) are designed to accommodate all of the cables and pipe penetrations, as well as to shield gammas and neutrons.

The major biological shielding includes the surrounding shielding walls, the TC upper shielding plugs, the PCPs, and the TC floor between the TC and the LS room.

**Fig. 8:**
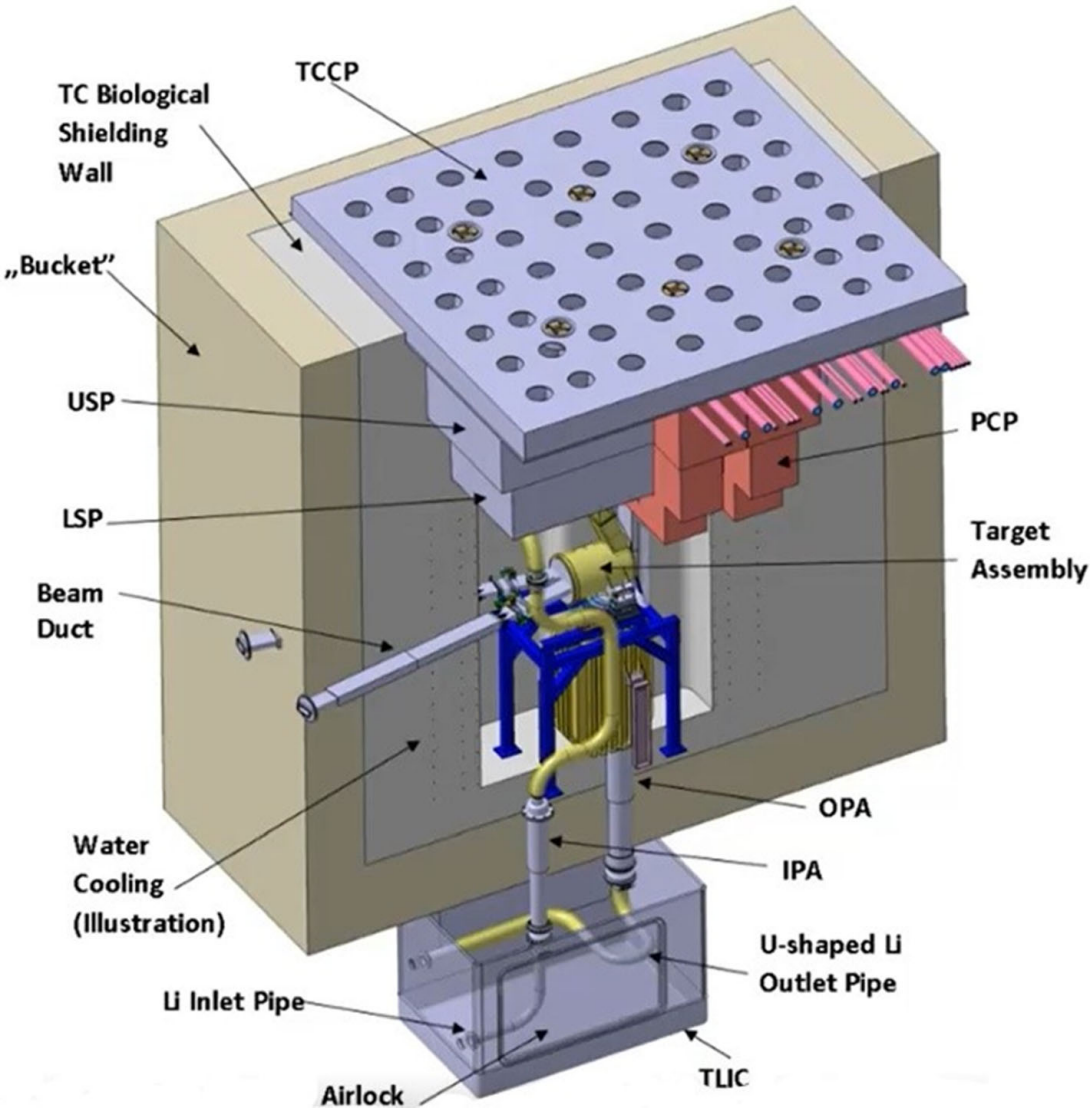
The IFMIF-DONES test cell (monolithic concept)

During the IFMIF/EVEDA phase, a monolithic concept of the TC was developed (Fig. 8). However, this concept turned out to be a weak point in the IFMIF-DONES design as the TC would be impossible to maintain in case of critical failure of the cooling pipes embedded in the shielding walls. Therefore, a new design, based on the so-called Maintainable Test Cell Concept (MTCC), was developed within the FP8 [34].

The MTCC is currently the reference configuration for the Test Cell. The main idea behind this concept is to produce a design where, in case of an unexpected failure of the liner and/or of the shielding walls, those components can be replaced by controlled and predefined procedures during a defined time.

**Fig. 9:**
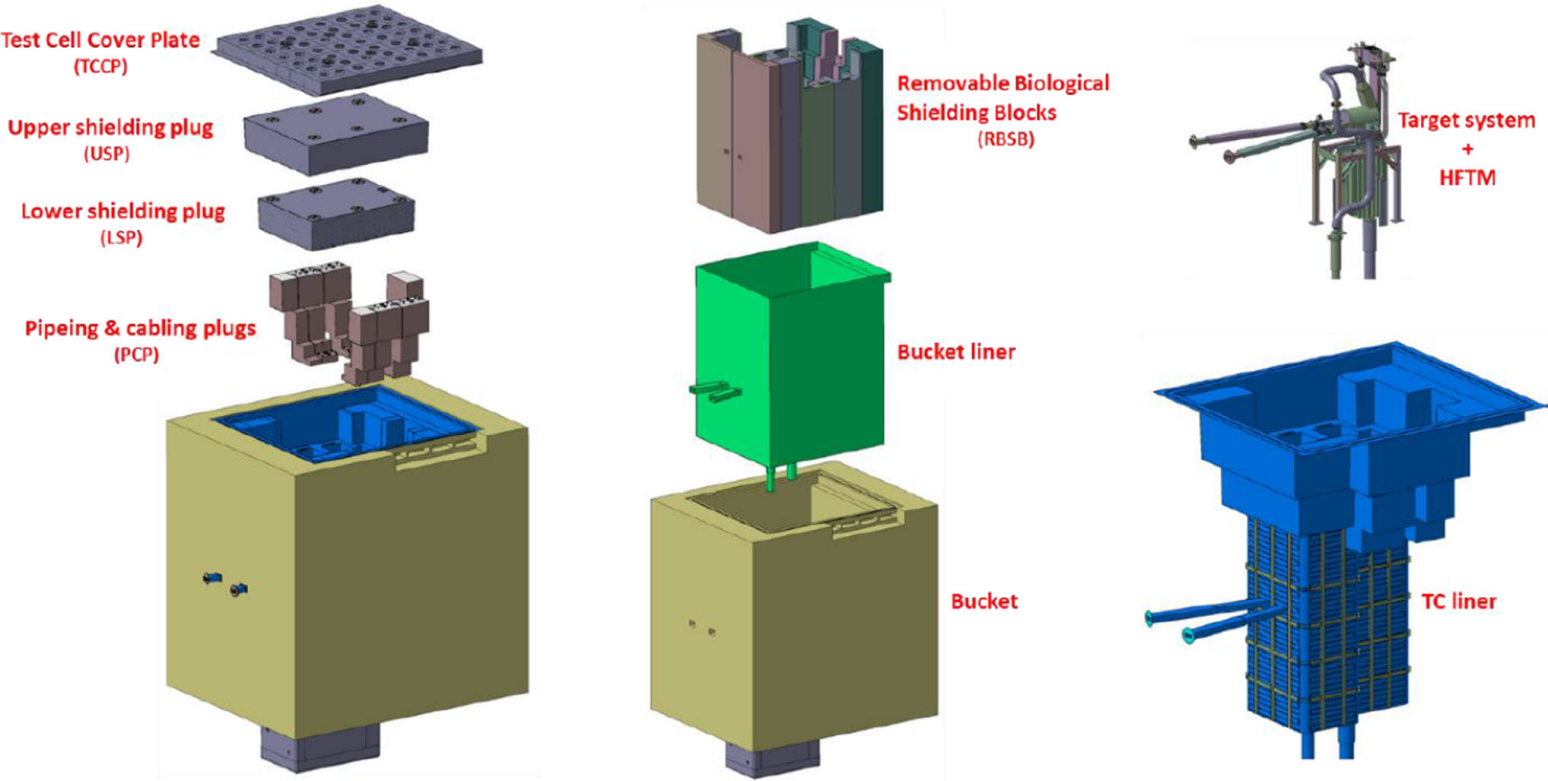
Exploded view of the current reference Maintainable Test Cell Concept (MTCC)

Figure 9 shows an exploded view of the MTCC, where the main components are identified. In comparison to the monolithic non-maintainable approach the main design change is that the inner concrete shielding which was non removable, has been substituted by eleven removable biological shielding blocks (RBSBs) of 1.5 m thickness. Another significant difference between the two concepts is that the Maintainable Test Cell Liner (MTCL) is no longer embedded in the surrounding concrete but is now made of a stand-alone piece which allows its full replacement. A preliminary mechanical analysis of this component has been reported in [35].

The RBSBs are located inside the so-called bucket, which is a permanent concrete structure built as part of the building, also shown in Fig. 9. The RBSBs and the bucket are separated by a permanent, 8 mm thick, non-cooled bucket liner which is permanently fixed to the concrete bucket.

The consolidation of the engineering design of the RBSBs and of the MTCL is currently being continued as part of the FP9 design activities.

#### The High Flux Test Module

**Fig. 10:**
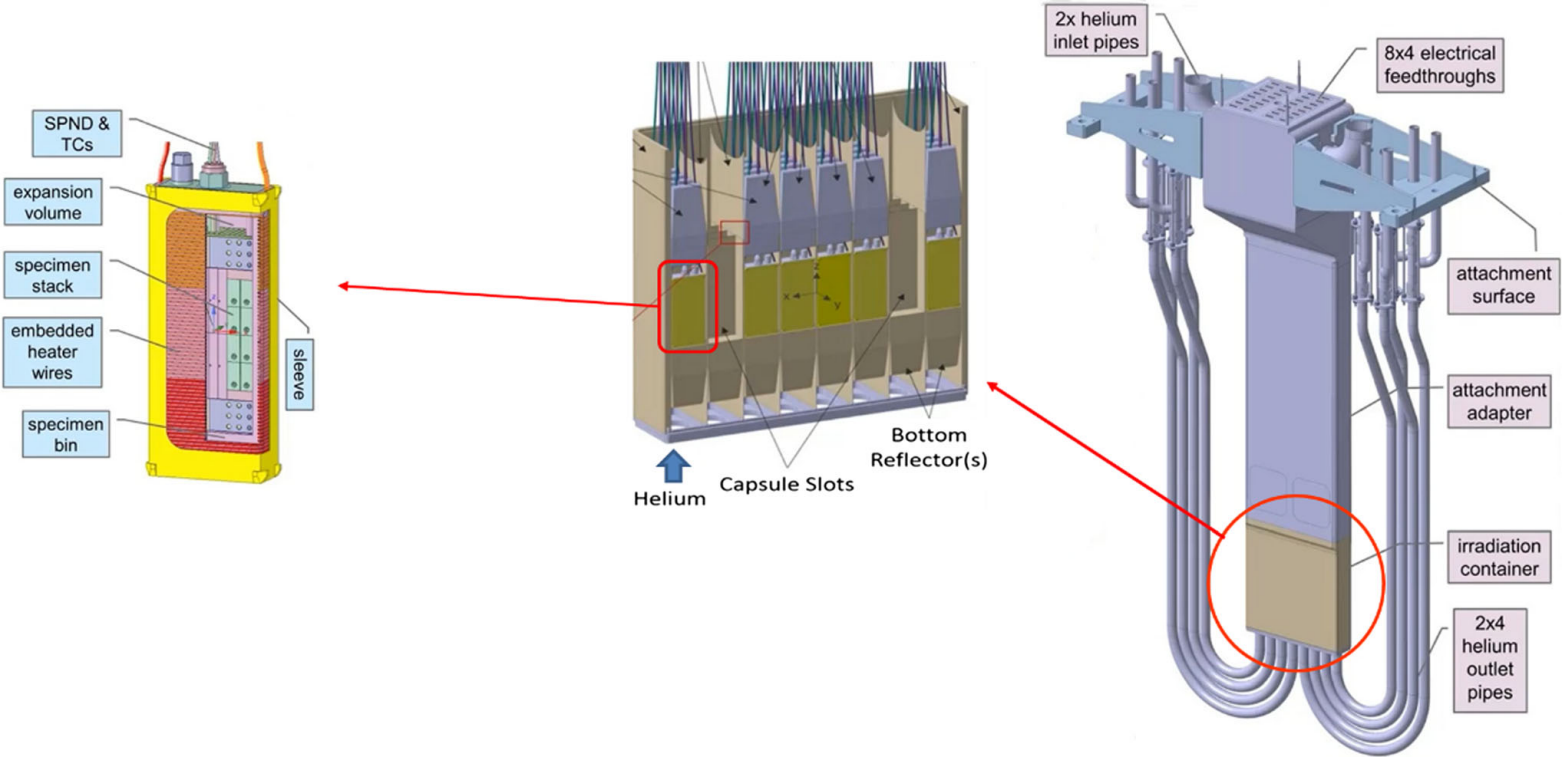
Design configuration of the High Flux Test Module (HFTM)

The basic configuration of the High Flux Test Module (HFTM) is illustrated in Fig. 10. The material specimen stacks to be irradiated in the test module are hermetically enclosed in an Irradiation Capsule Assembly. The capsules thereby are installed in the container in so-called compartment slots.

The temperature of the capsules set in each compartment can be selected independently among the values 250 °C, 350 °C, 450 and 550 °C through a series of electric heaters installed on each capsule and of separating gaps with different size (depending on the temperature) each filled with stagnant cooling helium. In addition, He gas is flowing inside mini-cooling channels of the HFTM container at variable (tuneable) conditions.

A total number of about 850 specimens can be irradiated at a damage dose rate of 12–25 dpa/fpy with gas production rates of about 13 appm(He)/dpa and 53 appm(H)/dpa [18]. The foreseen instrumentation includes several K-type thermocouples, activation foils, Self-Powered Neutron Detectors (SPND) and Micro Fission Chambers (MFC).

**Fig. 11:**
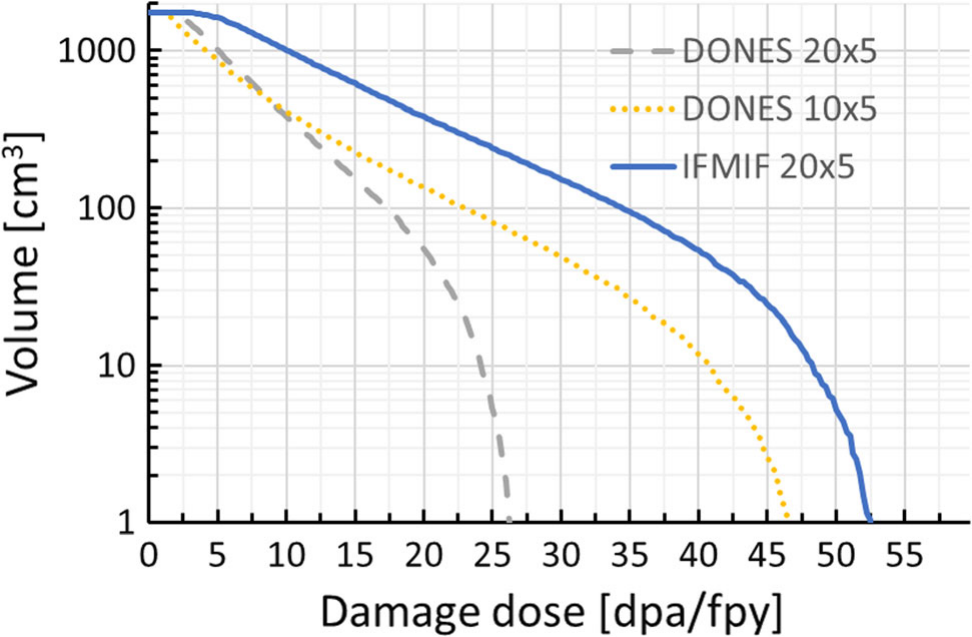
Damage dose versus irradiation volume performances in different cases

The performance achievable in terms of irradiation volumes versus damage dose is shown in Fig. 11 for the two (smallest and largest) IFMIF-DONES footprint configurations and also, for the sake of comparison, for the IFMIF case.

The engineering design of the HFTM has been largely consolidated during the FP8 also taking profit of the work done during the IFMIF/EVEDA phase. However, a number of activities still remains to be carried out to finalize the design especially concerning the validation tasks which include for example the test of critical manufacturing parts (e.g., the HFTM container) and the irradiation of sensitive components like the heaters and the electric connectors, to be conducted in the MARIA research reactor in Poland.

#### The Start-Up Monitoring Module (STUMM)

**Fig. 12:**
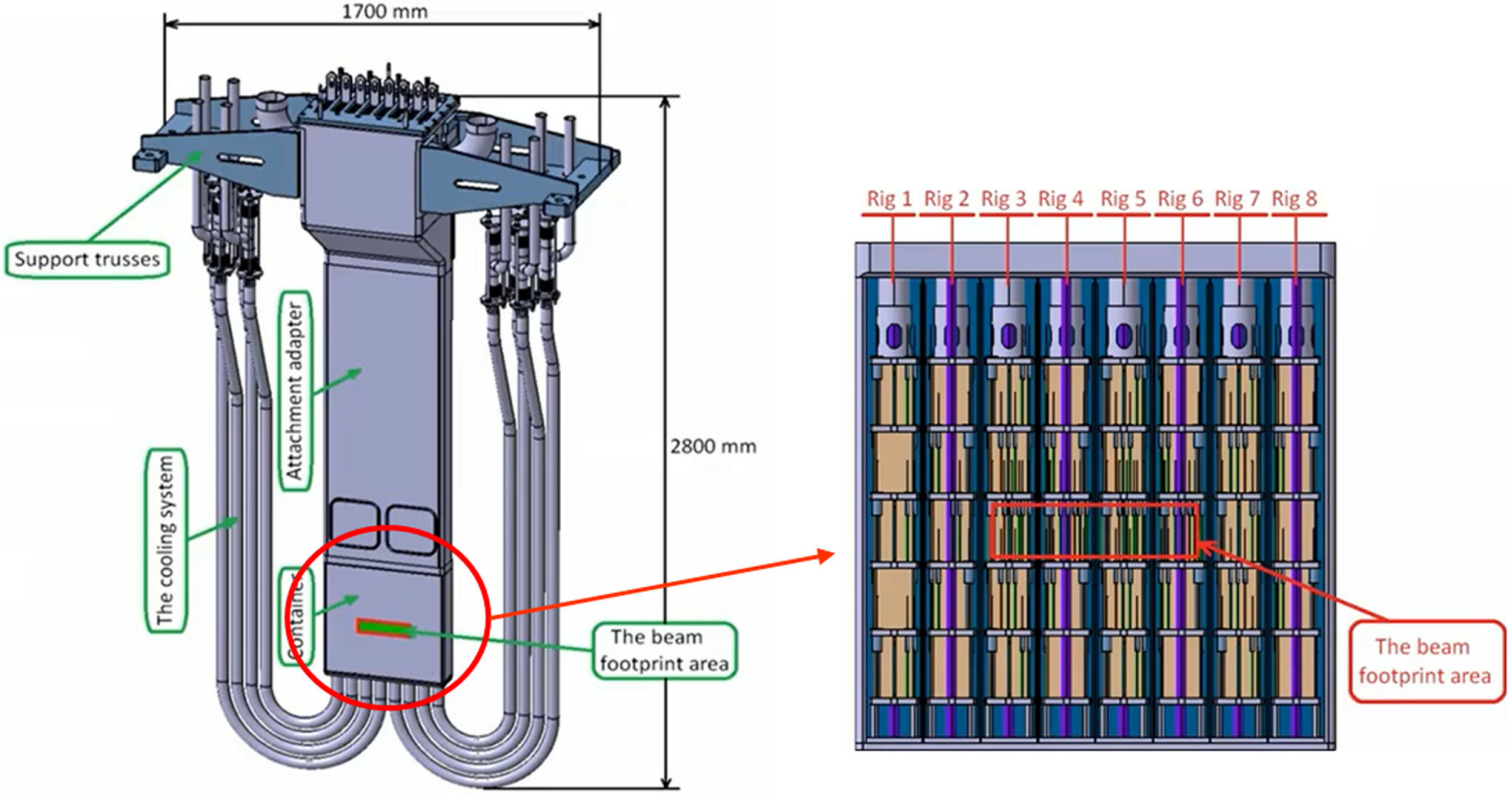
Configuration of the IFMIF-DONES Start-Up Monitoring Module (STUMM)

The STUMM is a fully instrumented module (Fig. 12) dedicated to the commissioning phase of IFMIF-DONES. It will be installed just behind the lithium target in the same position of the HFTM. Once the commissioning is completed, it will be replaced by the HFTM. The module can also be used when restarting after an irradiation stop to check that the proper neutron flux conditions at the samples positions are met.

The STUMM is designed with the function of characterizing the neutron and gamma fluxes at the HFTM position and of validating the neutronic models used to analyze the radiation field in the TC.

**Fig. 13:**
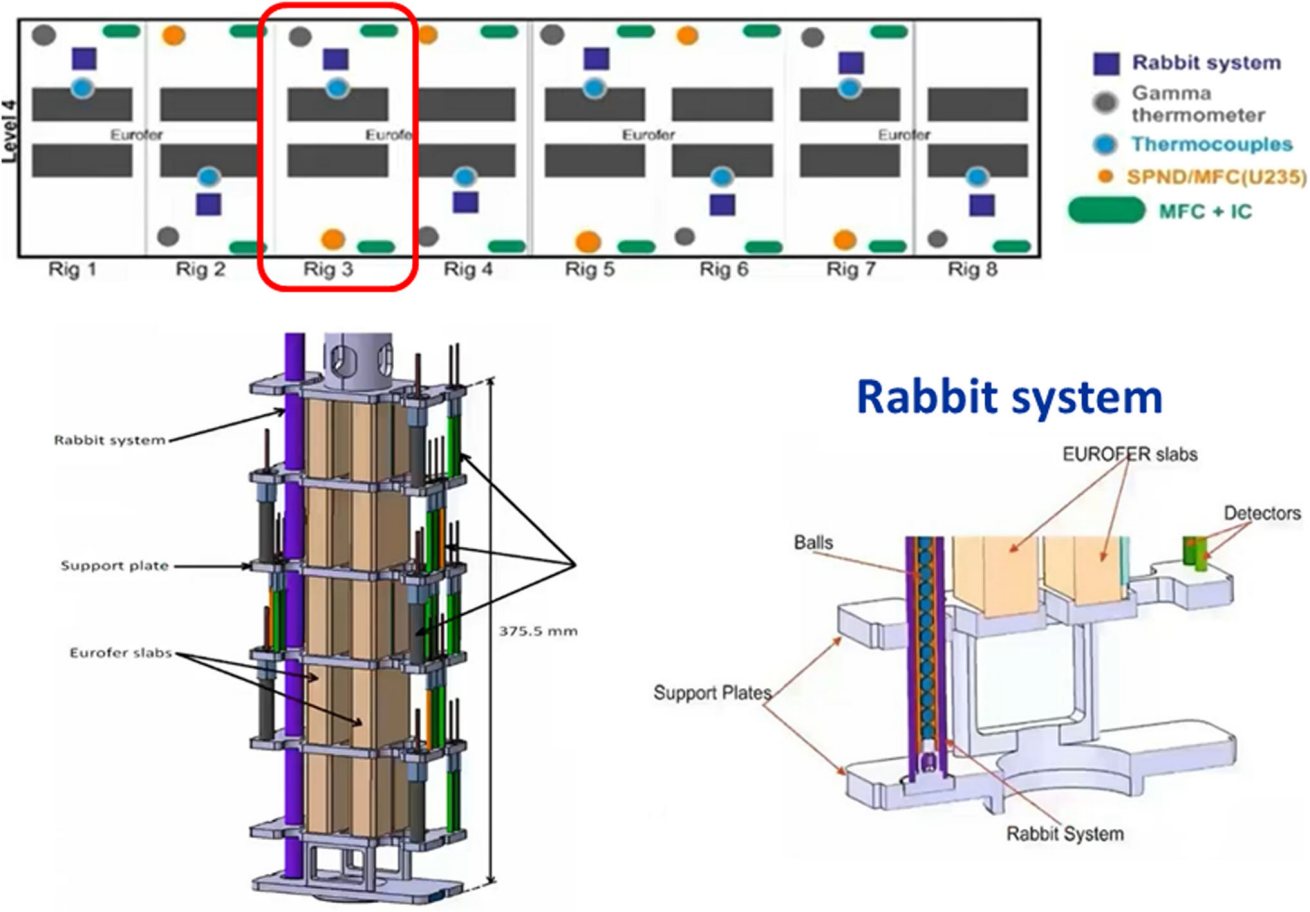
Instrumentation arrangement inside the STUMM

The following instrumentation is planned to be installed in the module (Fig. 13):

- SPNDs and/or MFCs with ^235^U for thermal neutrons flux measurements.
- MFCs with ^238^U for fast neutrons flux mesurements.
- ionization chambers.
- gamma thermometers.
- rabbit system with activation dosimeters.

During FP8 activities, the design of the STUMM has progressed approximately in parallel with that of the HFTM with which it shares many features (in fact, except for the inner part, the rest of the structure is quite similar for both systems) [36]. Most of the engineering design has been consolidated but significant work still remains to be accomplished especially for what concerns the integration aspects inside the TC and, more in general, with the other systems of the plant (for instance, the electrical and media connections with the PCPs and the interface with the rabbit system measuring station).

#### Facility for Complementary Experiments

IFMIF-DONES will be a powerful source of fast neutrons and, as such, it is of interest for various scientific and technology communities. Besides irradiation and validation of materials for the fusion program, it has been proposed to extend the scientific objectives of IFMIF-DONES by including in its design the Facilities for Complementary Experiments (FCE).

As part of the FP8 work, a White Book report on Complementary Scientific Programme at IFMIF-DONES [37], listing a collection of science cases for the complementary research has been prepared by a group of international experts. The interested areas include, in particular: (1) applications of medical interest, (2) nuclear physics and radioactive ion beam facility, (3) basic physics studies, and (4) industrial application of neutrons. The White Book demonstrates that many subjects and questions of today’s science in active fields of research could be investigated at IFMIF-DONES without compromising its main role as a material irradiation facility for the fusion programme. Following the conclusions of the report, different possible options for using (1) a fraction of the accelerated deuterons extracted from the D+ beam line and/or (2) the neutron flux still available behind the HFTM, have been proposed to be possibly incorporated into the baseline configuration of the IFMIF-DONES facility.

**Fig. 14:**
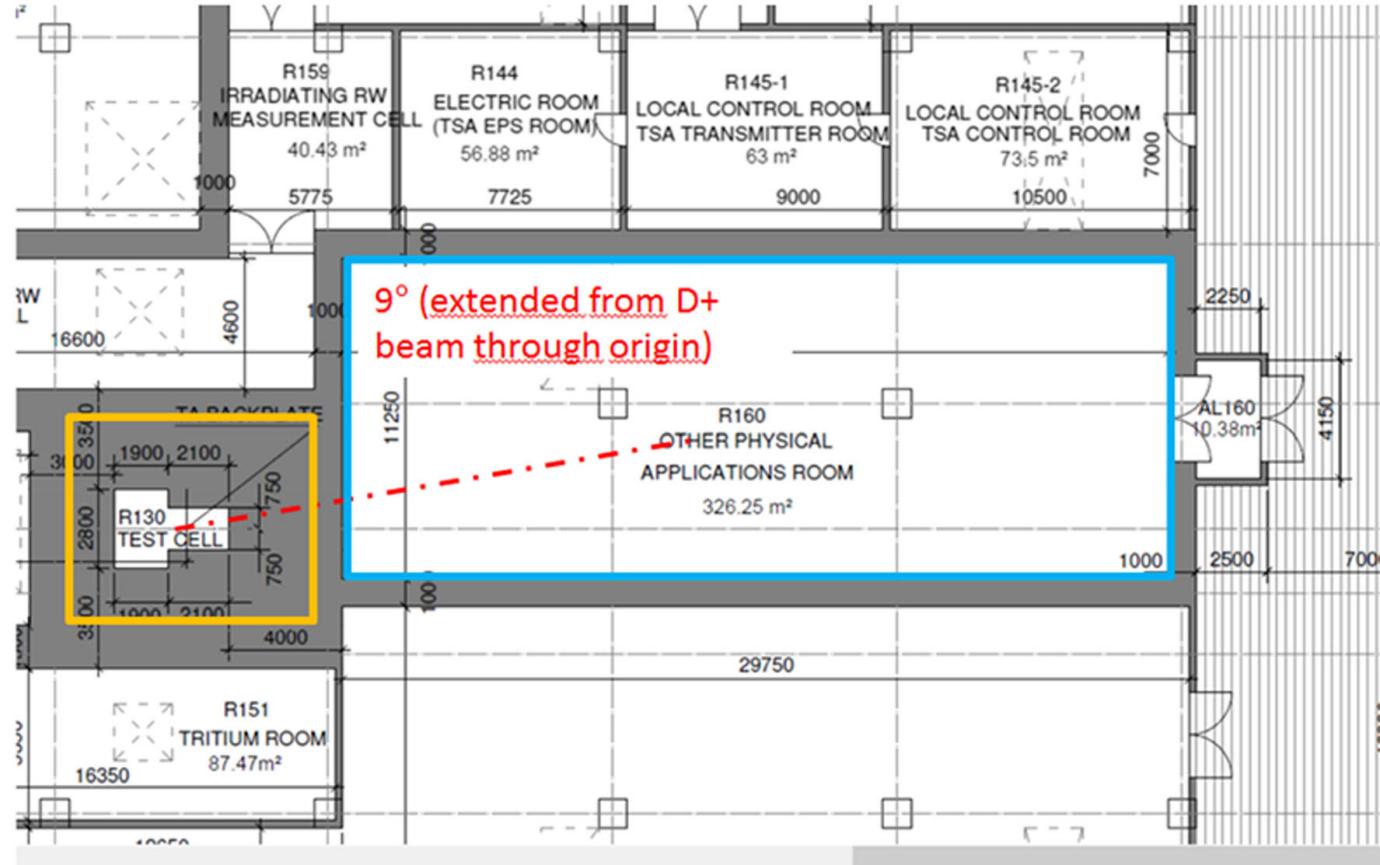
Room for Complementary Experiments with neutrons behind the TC

Presently, only the option (2) has been implemented, at a conceptual level, in the design of the facility. A dedicated room adjacent to the TC (Other Physical Applications Room in Fig. 14) has been reserved. Neutrons will be extracted from the TC and delivered to this room by means of a neutron beam shutter embedded in the shielding wall separating the two areas. The beam shutter will allow independent operation in the complementary experiments room without affecting the irradiation of the samples in the HFTM. Moreover, it will optionally offer the possibility of moderation of the neutron flux as required by the experiments.

The next steps to be performed in the FP9 will be focused on the detailed design of the neutron transport line including the neutron beam shutter and on the feasibility study of experiments using an extracted D+ beam fraction from the main beamline.

### Site, Building and Plant Systems

#### Main Building and Plant Systems

**Fig. 15:**
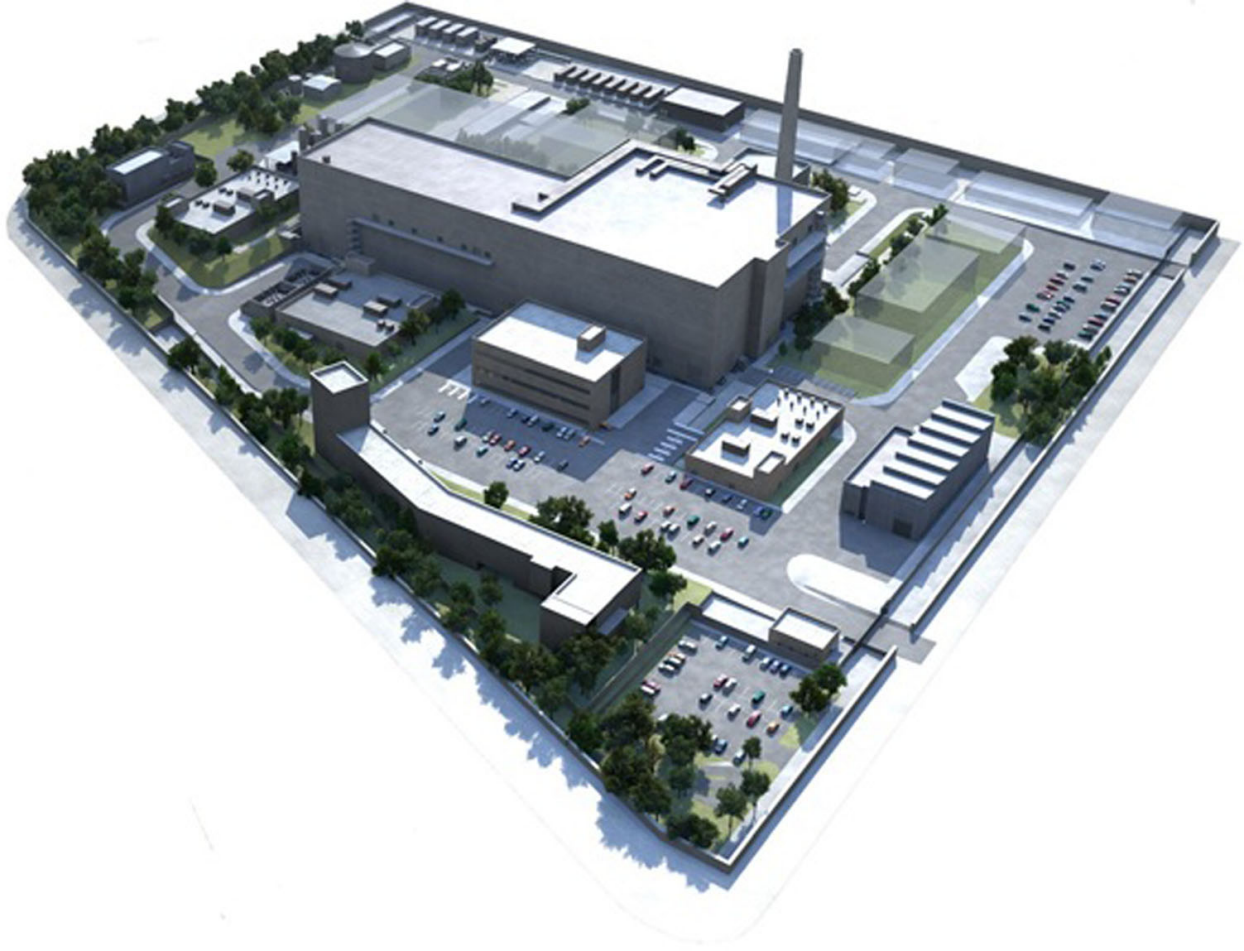
Overview of the IFMIF-DONES site with the MB at the center

The IFMIF-DONES Main Building (MB) is the central building housing all the major systems of the facility. It is a four-story building with footprint dimensions of about 160 m x 75 m and a height of some 32 m. It is erected in the middle of the site with many auxiliary buildings (such as, for instance, the electric power substation, the warehouse, the cooling towers, etc…) arranged around it (Fig. 15). It is designed with a seismic isolation pit, taking also into account space reservation for a possible future upgrade to the full IFMIF configuration (through the integration of a second accelerator).

The design of the IFMIF-DONES Main Building (MB) has evolved from a very preliminary stage to the present status. The allocation of space and rooms in the MB has been reviewed and detailed. A significant external milestone of the project took place in 2017 when the Escuzar site near Granada in Spain was selected as the reference location for the construction of IFMIF-DONES. This selection has been made based on the proposals expressed by the EU countries interested in hosting the facility (Spain and Croatia) and the positive evaluation by the Fusion for Energy (F4E) Governing Board in its 2017 December meeting of the joint Spanish-Croatian proposal to support the candidature of Granada. Although the decision to start the construction of IFMIF-DONES has not been taken yet, from that date on (i.e., since 2018) all the design activities of the WPENS work package have been carried out assuming that, from the technical point of view, IFMIF-DONES will be built in Escuzar. Consequently, the MB configuration was reviewed and adapted to the specific site characteristics with the key modification of incorporating a seismic isolation concept for the Main Building. The basement was extended to cover the whole surface footprint of the building, so providing a uniform horizontal plane of support to the building itself that could benefit the design of the seismic isolation system. Contrary to the former IFMIF-DONES design in which the Main Building was supposed to be located partially underground, it was decided to fully relocate it above ground surface. As a consequence, the seismic isolation pit depth was reduced to the minimum, improving earthwork balance. Some preliminary activities were carried out for the seismic characterization of the site as well as for the preliminary environmental impact studies. Due to the plot characteristics it was decided that the IFMIF-DONES site will be terraced, with different elevations instead of being a flat plot. The studies determined the location of all of the Auxiliary Buildings on the IFMIF-DONES site.

Along with the definition of the Main Building also the functions and the design of the Plant Systems providing all the common services to the facility were detailed. These systems include the HVAC (Heating, Ventilation and Air Conditioning) System, the Electrical Power System, the Heat Rejection System, the Service Water System, and the Service Gas System as well as the Solid, Liquid and Gas Radioactive Waste Treatment Systems and the Fire Protection System.

#### Remote Handling

**Fig. 16:**
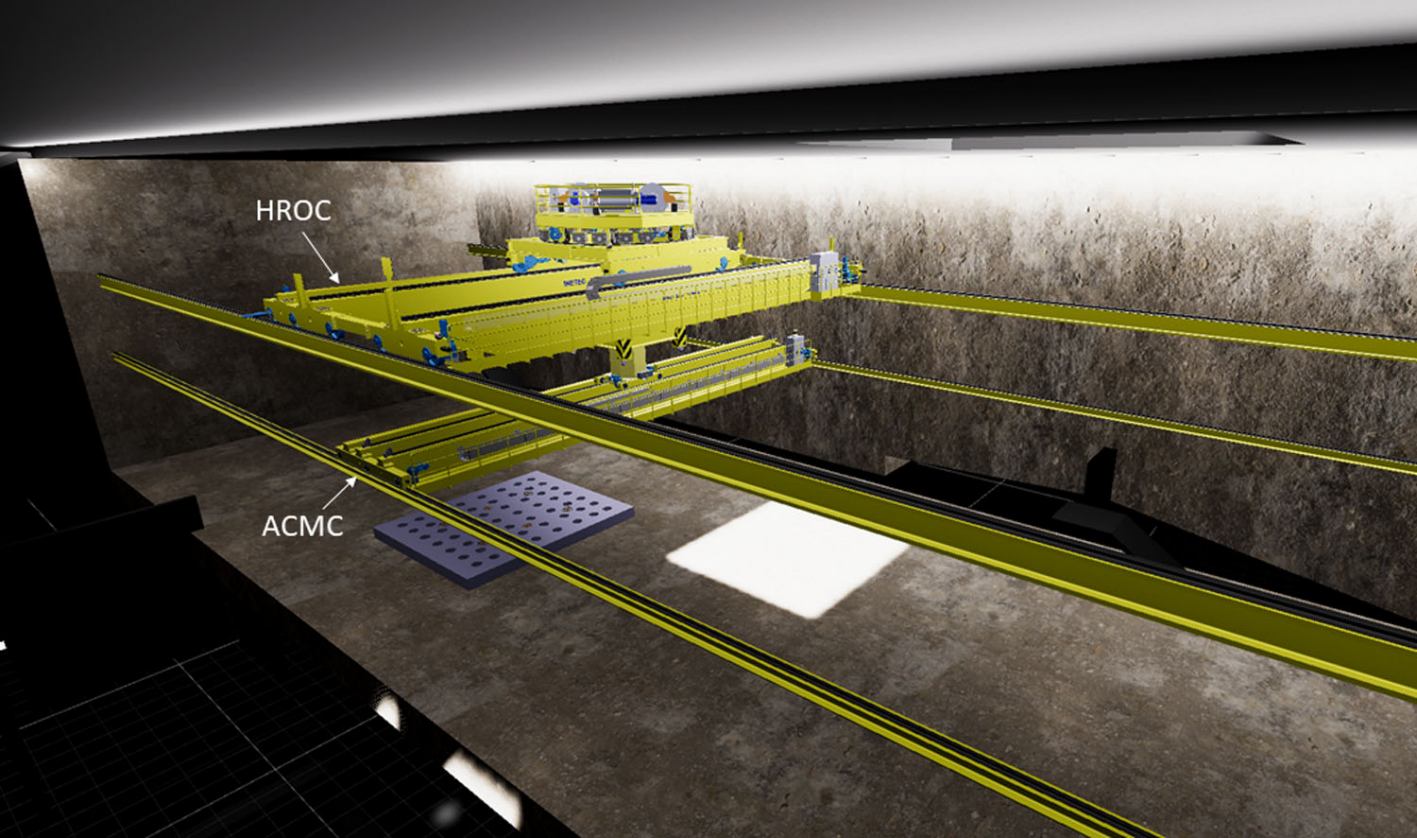
View of the AC with HROC and ACMC installed

**Fig. 17:**
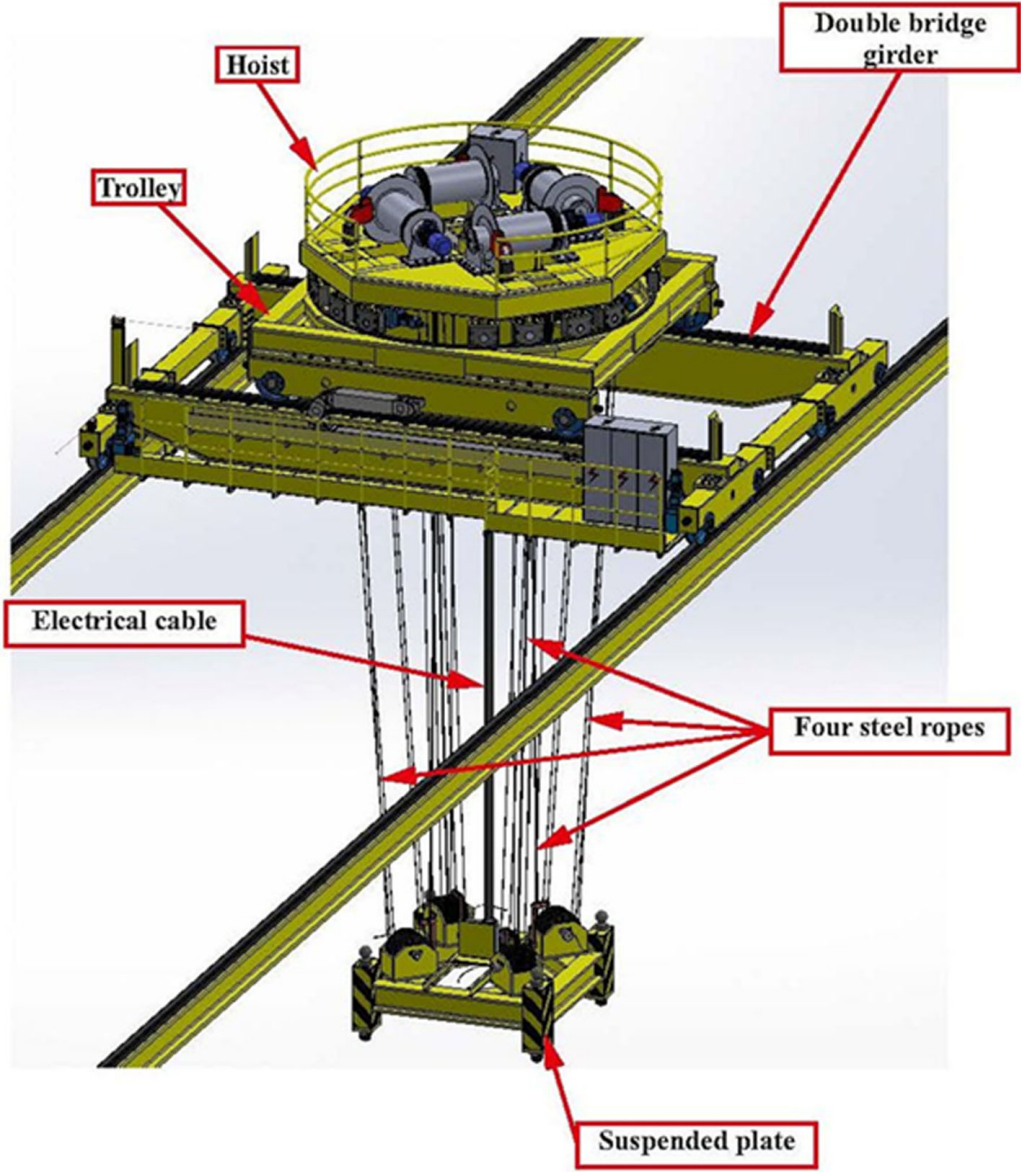
Heavy Rope Overhead Crane (HROC)

**Fig. 18:**
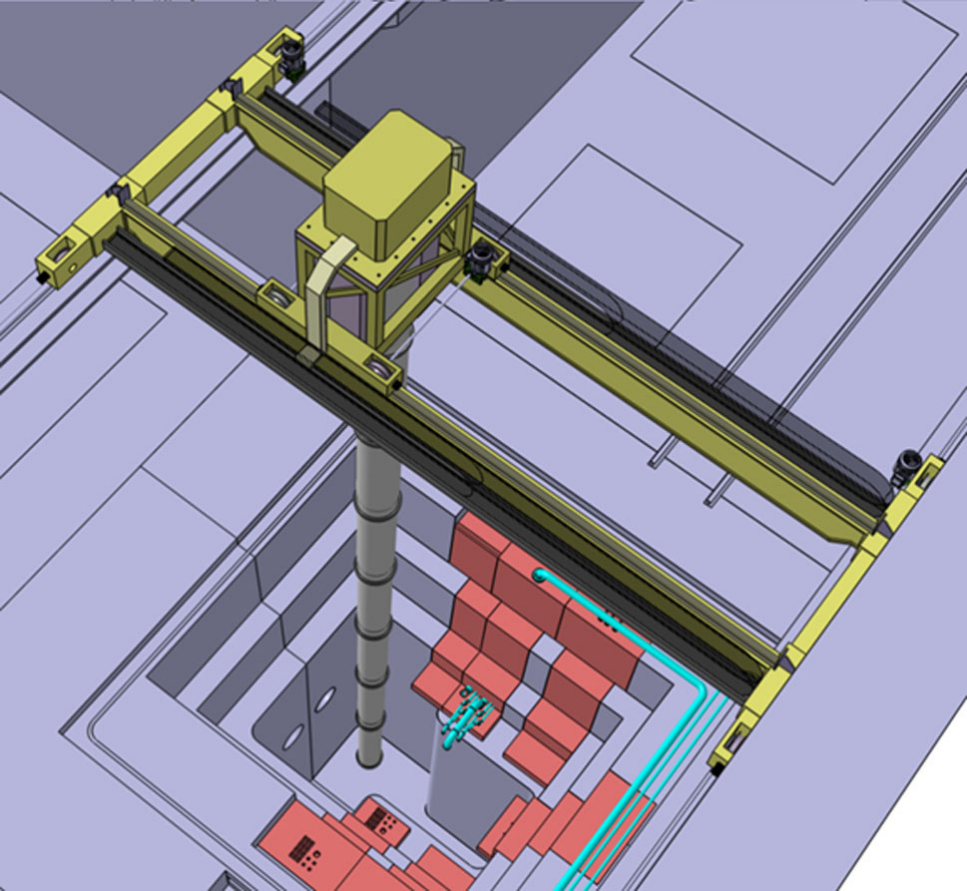
ACMC with its telescopic mast operating inside the TC

Due to the strong radiation environment, the maintenance of equipment located in the TC and in adjacent areas such as the Target Interface Room (TIR) and the Irradiated Materials Treatment Cell (IMTC) as well as of some equipment in the accelerator vault, will be performed through Remote Handling (RH) means. In particular, the most demanding RH operations will be performed from the Access Cell (AC) which is located above the TC. The AC is equipped with two cranes (Fig. 16):

1. The Heavy Rope Overhead Crane (HROC, Fig. 17) which is a nuclear-grade multi-ropes double beam overhead travelling crane with a payload capacity of 140 tons, designed to perform transfer operations of the heaviest components (i.e., weighting several dozens of tons), like the Upper and Lower Shielding Plugs (USP, LSP) or the TC Cover Plate (TCCP).
2. The Access Cell Mast Crane (ACMC, Fig. 18) which is a nuclear-grade double beam overhead crane equipped with a telescopic mast with a payload capacity of 2 tons, to support and locate manipulators at the correct position in the TC thus allowing RH operations like for example replacement of TAA and HFTM, cleaning, inspection, bolting, etc….

The telescopic mast of the ACMC is equipped with a common interface (the so-called Gripper Change System, GCS) that allows a safe and fast exchange of the RH tools.

In addition to those performed in the AC, other RH operations are foreseen in the Li loop area and in the high-energy section of the accelerator by means of dedicated equipment.

At present, the design maturity level of the various RH systems is quite different across the several areas of the facility: some of them are currently in a very advanced state (e.g., the HROC and the ACMC which are almost completed and ready for tendering; or the RH tools for the TAA replacement) while others are less advanced or even still in a conceptual phase (e.g., the RH systems for the LS).

### Central Instrumentation and Control Systems

An important part of the FP8 activities was the definition and the preliminary design of the Central Instrumentation & Control Systems including the CODAC System; the Machine Protection System; and the Safety (Control) System [38]. This work included discussion and definition of systems architecture and of their interfaces with the Local Instrumentation & Control Subsystems (LICS). During 2020, a review of the roles and functions of the Central Control Room (CCR) was performed with the goal to better define its requirements and confirm its location inside the Access Building connected to the Main Building.

### Transversal Activities: Project-Level Analyses

Some transversal activities grouped as “Project-Level Analyses” include studies on Safety, Neutronics, RAMI (Reliability, Availability, Maintenability and Inspectability) and Logistics and Maintenance. They also have reached a good level of maturity during the FP8 period as succinctly explained in the following paragraphs.

Safety has been the driver for the design following the analyses of design-basis accidents scenarios. The critical Postulated Initiating Events have been studied and the classification of structures, systems and components in terms of Safety Important Class (SIC) equipment was performed and included in a Preliminary Safety Analysis Report (PSAR).

Neutronics has provided the information required for the assessment of shielding features of the different parts of the building; for the choice of the materials able to ensure the allowable dose rates and for the classification of the rooms based on their contamination and radiation levels.

RAMI has analyzed the critical components from the point of view of availability, carrying out FMECA (Failure Mode, Effects, and Criticality Analyses) and prompting changes in the design, mainly in terms of redundancy of some equipment.

Logistics and Maintenance has focused on the analysis of the flow of materials, proposing the required modifications in the main building for the feasibility of the replacement of equipment. Also the Maintenance Management Plan was issued during this period.

In addition, dedicated computer tools were provided by Project Management in order to implement a full Systems Engineering approach, thus facilitating the project integration. Management of interfaces and requirements started to be done through web-based applications, enhancing the exchange of information.

Some preliminary studies concerning the decommissioning plans of the facility have also been carried out [39]. The decommissioning assessments will in fact be part of the licensing documentation to obtain the operation permit from the Regulator.

## Validation Results and Lessons Learned

A number of important validation results and lessons learned relevant to the design of IFMIF-DONES has been achieved both in the past IFMIF/EVEDA phase [40] and within the more recent EUROfusion WPENS (FP8) activities [41].

Concerning the IFMIF/EVEDA results, three main prototypes, among others, have been built and used for validating different aspects. These are, in particular:

- The Linear IFMIF Prototype Accelerator (LIPAc) which is presentlyunder commissioning at Rokkasho, Japan, cloning the IFMIF components up to its first superconductive accelerating stage (9 MeV, 125 mA of D+ beam in CW operation). Tiered-commissioning of the accelerator systems started in November 2014 and is planned to be completed by integral commissioning and long-pulsed operational testing in the next few years [42];
- The Experimental Lithium Test Loop (ELTL) built at Oarai, Japan [43], integrating all the elements of the IFMIF lithium target facility. It was commissioned in February 2011 and the test programme was completed in 2014 with the successful proof of the long-time stability of the liquid lithium jet forming the target [40, 44], complemented by corrosion experiments performed on the Lifus 6 lithium loop constructed at ENEA Brasimone Research Centre in Italy [45];
- The HFTM prototype along with a prototype of the specimen capsules. This latter prototype was tested under irradiation in the BR2 fission reactor at SCK/CEN in Belgium but the results left some open questions regarding the performance under irradiation of the installed electric heaters which thus need to be finally addressed again in FP9. The HFTM prototype was successfully tested in 2015 under IFMIF design conditions in the helium loop HELOKA at KIT, Karlsruhe (Germany) [46]. This was complemented with the Creep Fatigue Test Module [47] manufactured and tested in full scale at PSI, Villigen in Switzerland [44].

Besides these, many other additional validation activities in various aspects have been carried out as part of the IFMIF/EVEDA project. The outcomes of these activities have been used as an input for the design of IFMIF-DONES together with the new experimental results obtained in the framework of the WPENS (FP8) work. The most important of the latter ones are described hereafter for each systems area.

### Accelerator Systems Validation

#### LIPAc Commissioning Tests

Validation activities for the Accelerator Systems were mainly performed on LIPAc. Although not part of WPENS, these activities are strictly linked to the IFMIF-DONES design as the validation of the accelerator performance is one of the most critical points in the development of the facility.

**Fig. 19:**
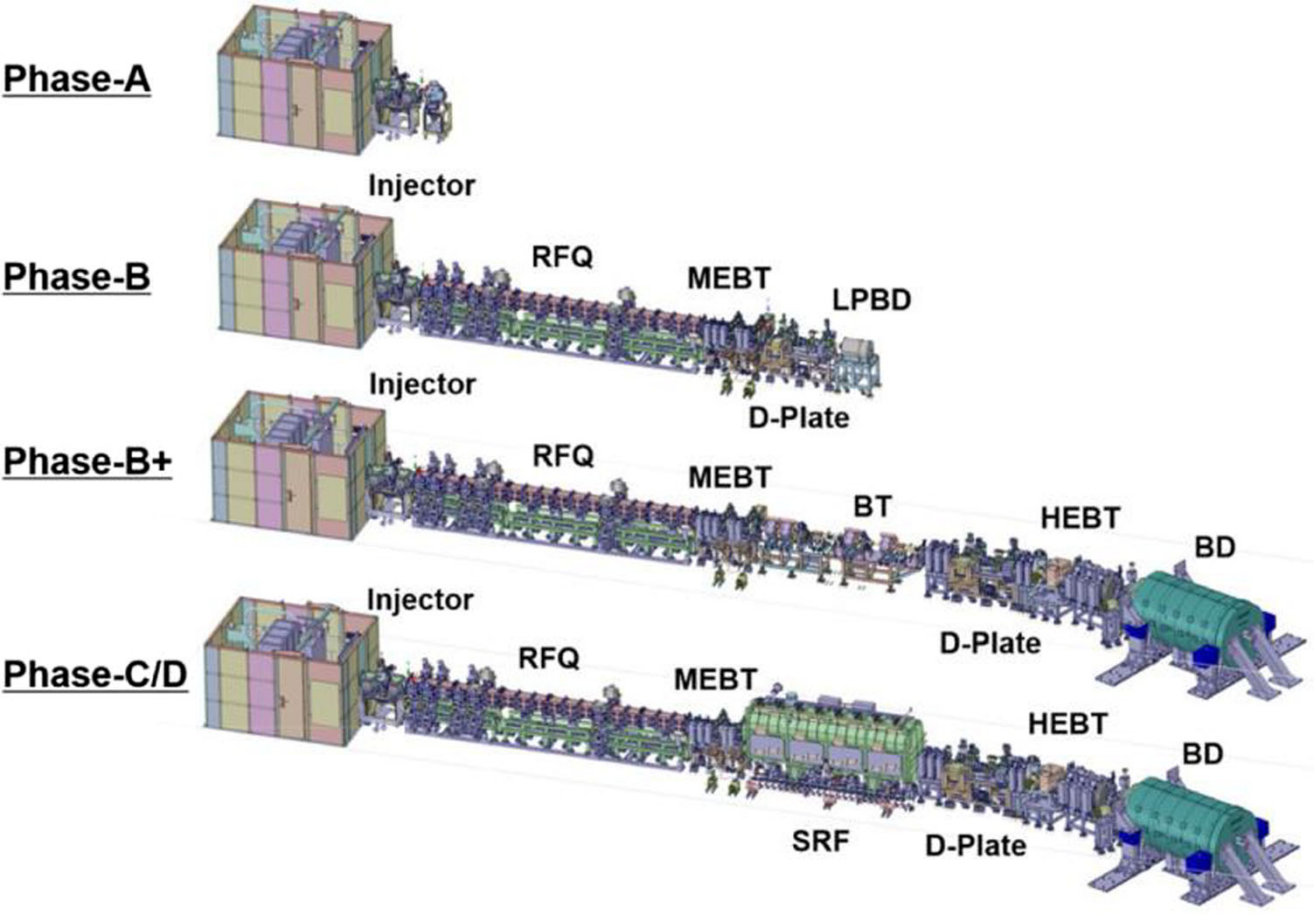
Configuration of the LIPAc prototype during the different commissioning phases

The LIPAc accelerator is presently being commissioned in several phases (Fig. 19). The injector performance was confirmed in the experimental campaigns of the so-called phase A during 2015 and 2016. At low duty cycle operation (below 5%), the ion source has been shown to be stable and reliable while extracting a 155 mA total beam.

**Fig. 20:**
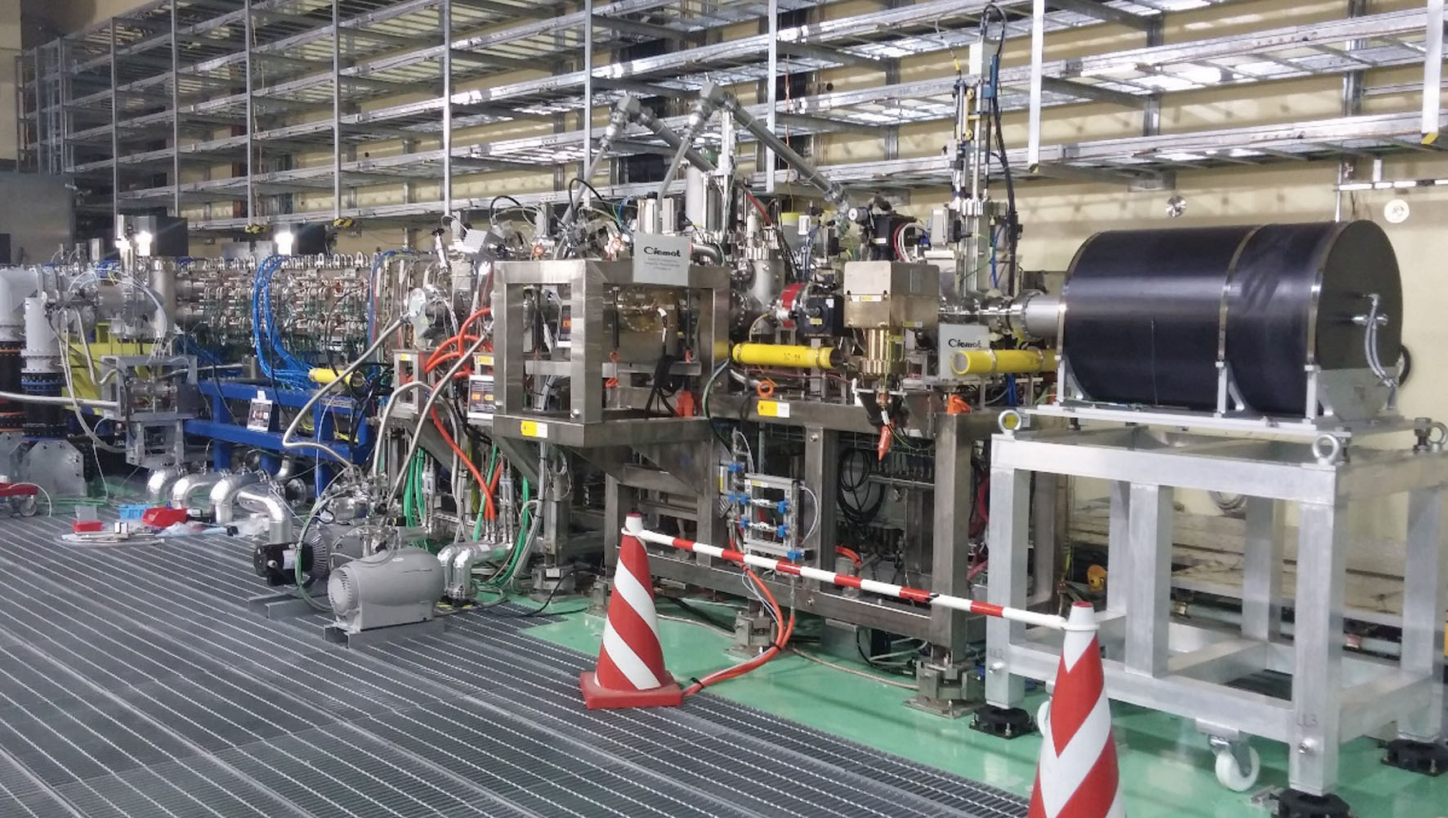
LIPAc configuration during Phase B commissioning stage

The successive Phase B included the deuteron beam acceleration through the RFQ up to 5 MeV in pulsed mode at low duty cycle (Fig. 20). This phase was finally completed in July 2019 with the successful injection of a 140 mA D+ beam with a duty cycle of 0.1% into the RFQ, leading to a 125 mA, 5 MeV accelerated beam at its exit [29]. Under these conditions, the RFQ transmission was 90%, in line with what was expected and the beam was characterized and losslessly transported to the aluminum low power Beam Dump. This represents the most relevant result achieved so far for what concerns the acceleration technology validation, showing that the IFMIF-DONES accelerator concept seems feasible and no evident showstoppers are in place.

The next Phase B+ which involves the 5 MeV deuteron beam acceleration with progressive increase of the duty cycle up to full power continuous beam is presently being prepared. Finally, phase C includes the test of the whole accelerator including the SRF Linac, producing therefore a continuous deuteron beam of 9 MeV energy.

### Lithium Systems Validation

#### Erosion/corrosion Tests in Flowing Li

**Fig. 21:**
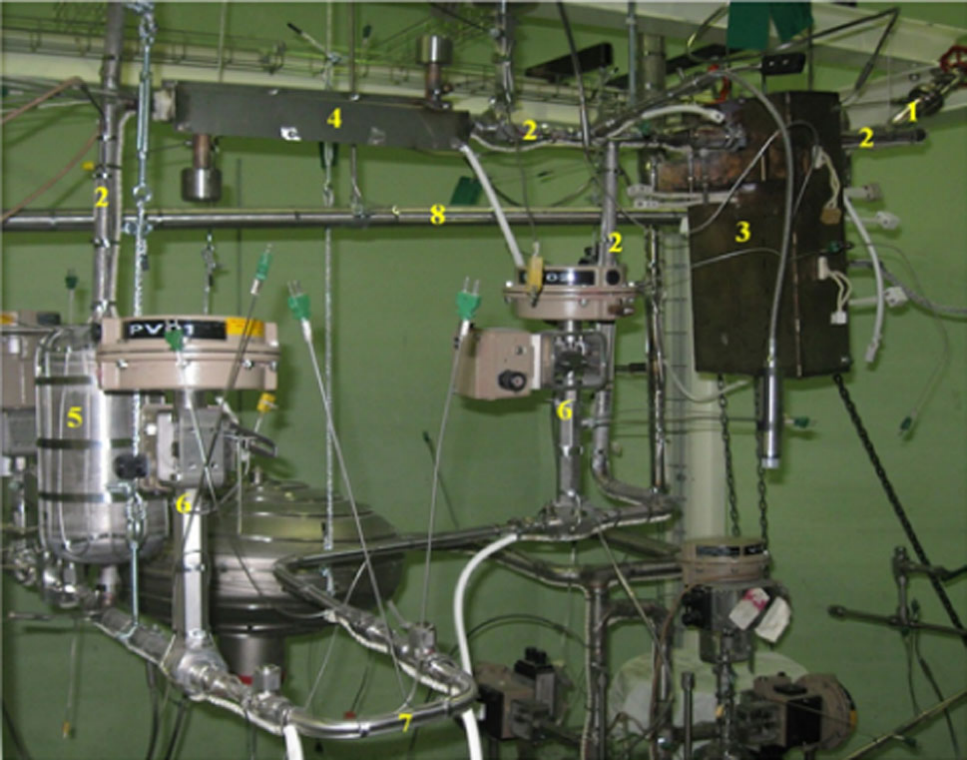
Lifus 6 loop at ENEA Brasimone Centre

An important validation activity for the LS concerns the assessment of the steel corrosion in the Li loop and in the Target Assembly. New corrosion tests were carried out within the FP8, following the ones already performed during the IFMIF/EVEDA phase. The facility employed for this purpose was again Lifus 6 (Fig. 21), a liquid lithium plant designed and realized at the ENEA Brasimone Research Centre in Italy.

The tests have been conducted on the Reduced Activation Ferritic/Martensitic (RAFM) steel Eurofer-97 and on the stainless steel AISI 316L (which are the reference materials for the TSY and the Li loop piping, respectively) at the relevant operating conditions of the system (Li temperature 330 °C; Li velocity on the specimen surface: 15 m/s) [48].

The main results achieved so far, from both IFMIF/EVEDA and WPENS tests, have shown that at low nitrogen concentration in the lithium (i.e. below 30 wppm) the corrosion rate of the EUROFER meets the TSY requirements (< 1 μm/year) while at higher concentrations (above 320 wppm of N) the corrosion rate is not acceptable neither for the EUROFER nor for the stainless steel, although the performed tests for the latter material are not relevant as the Li velocity in Lifus 6 test section was higher than that envisaged in the LS piping [41].

Further tests are thus planned to be performed in FP9 for different operating conditions (intermediate N concentrations, lower Li velocity, etc…) in order to expand the matrix results and more closely approach the real operating parameters.

#### Pre-heating Tests of the Target Assembly

Another important aspect in the LS validation is concerned with the pre-heating of the TAA during the start-up operation which is performed before injecting the lithium. The main objective was to demonstrate that it is possible to reach a minimum temperature in the Backplate conservatively higher than the lithium melting point (180 °C) without any active heating on it (since heaters cannot be used on the Backplate due to space restrictions) while ensuring the lowest possible temperature gradients within the rest of the structure in order to minimize the thermal stresses.

**Fig. 22:**
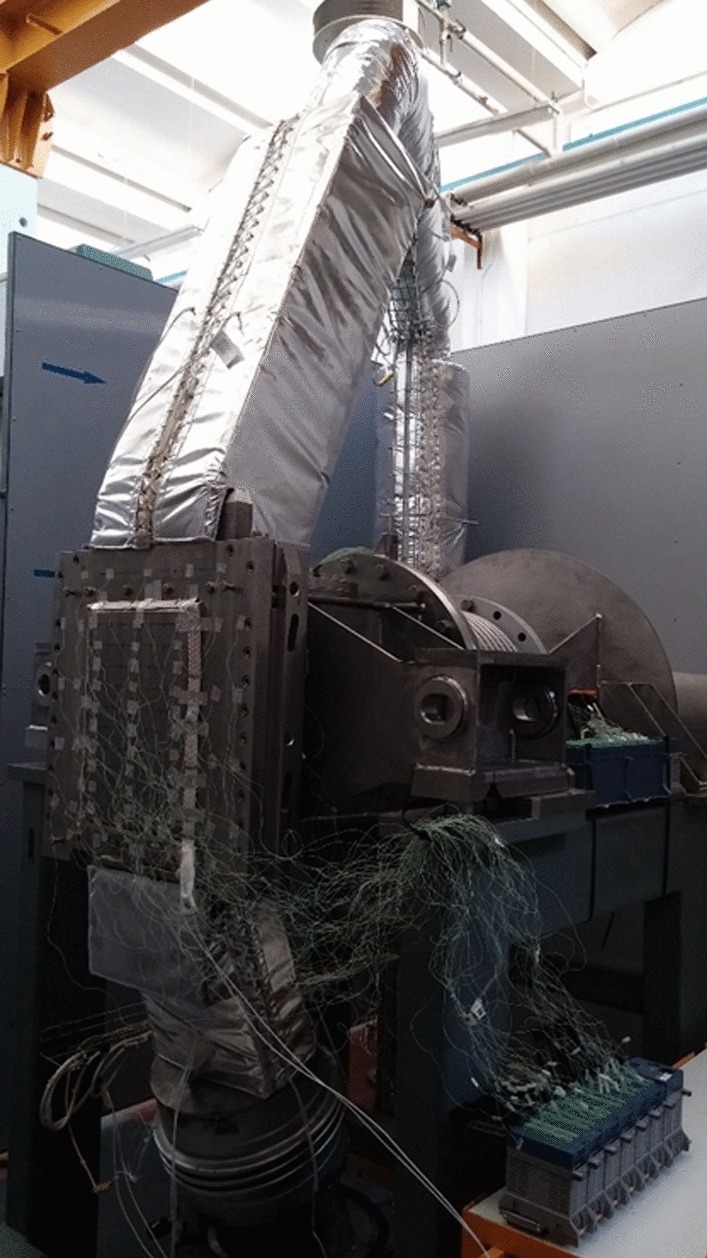
Pre-heating tests on the TAA prototype at ENEA DRP laboratory

To this purpose, a dedicated experimental activity has been carried out on the prototype of the former IFMIF Target Assembly already installed at the ENEA Brasimone DRP laboratory, using purposely designed heating jackets which cover the double function of heating and insulating the TAA structure (Fig. 22) [41]. In spite of some differences between the current IFMIF-DONES TAA configuration and the one adopted for the experimental campaign, it is expected that the obtained results are not significantly affected by these differences and that they are still relevant for the current TAA design.

The experimental results have shown that with the proposed TAA heating jackets configuration, it is possible to reach the minimum Backplate temperature required (> 200 °C) before injecting the lithium.

### Test Systems Validation

#### HFTM Validation

**Fig. 23:**
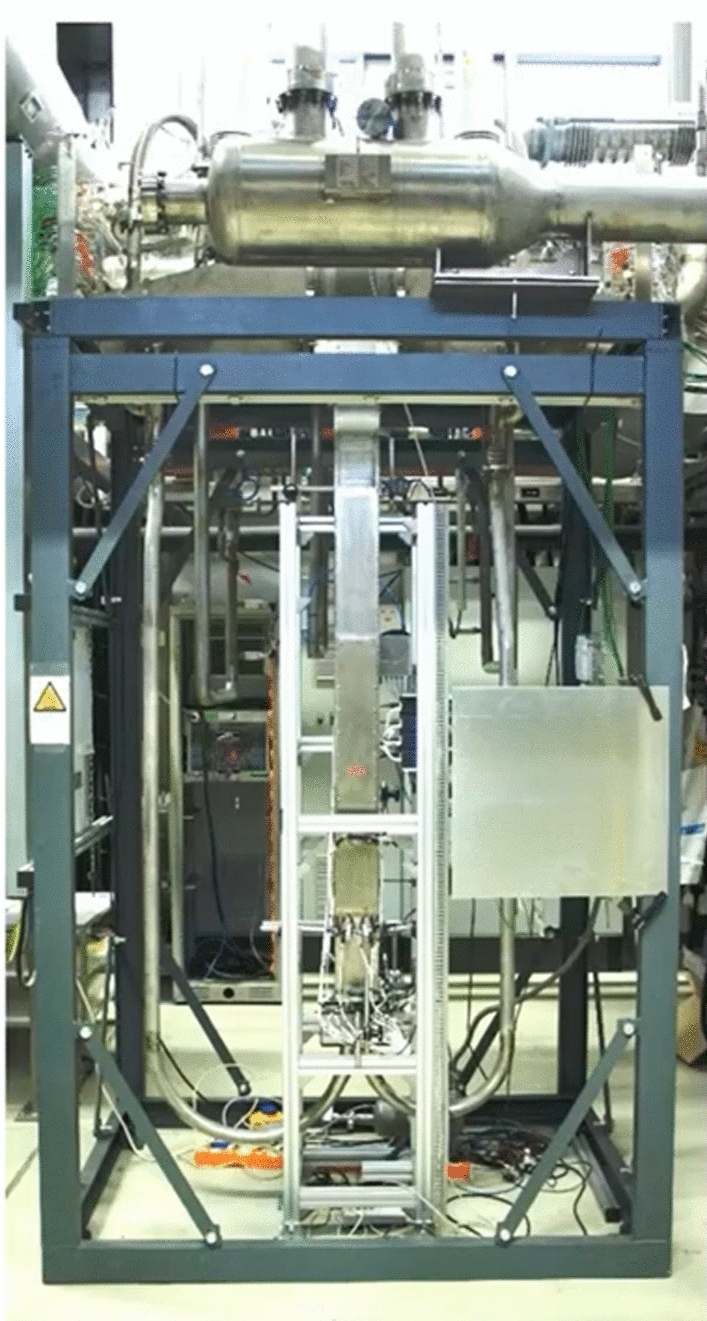
Testing of the HFTM prototype in the HELOKA loop at KIT (Germany)

The HFTM prototype was tested in the HELOKA helium loop (Fig. 23) in a first campaign in 2014–2015 for the full range of operating conditions, including pressure, mass flow rates, (surrogate) nuclear heating and capsule temperatures 250–550 °C. The full temperature range was achieved during the experiments [46].

The tests in the HELOKA helium loop confirmed the excellent thermal performance predicted by the design analyses in the operation temperature range 250–550 °C, with very low temperature spreads in the specimen payload and agile transient control behaviour. Stresses and vibrations in the HFTM structure were well below a critical level.

**Fig. 24:**
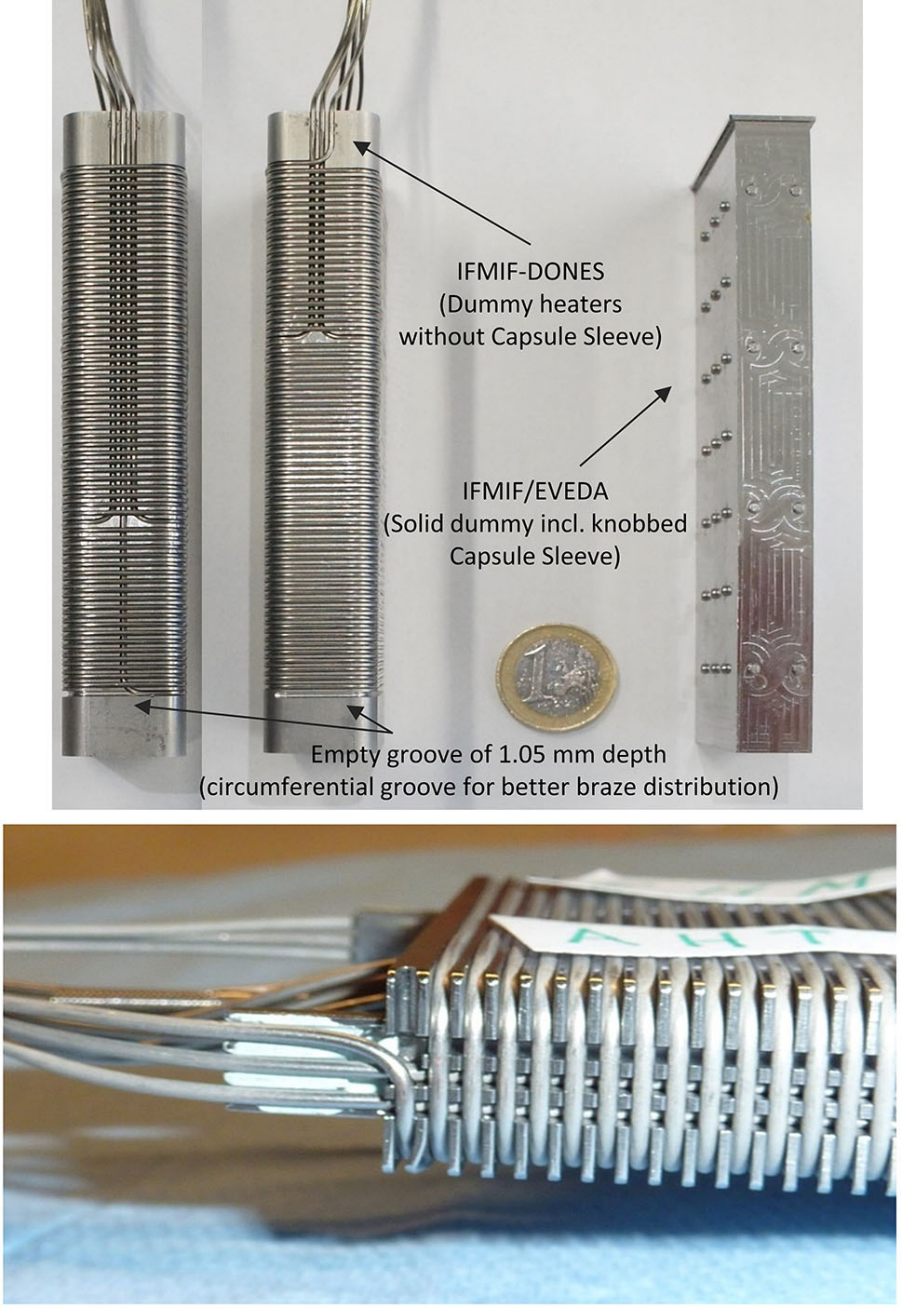
HFTM capsule prototypes with electric heaters

In 2020, new tests were carried out within the FP8 WPENS work programme, including three new fabricated irradiation capsules (Fig. 24).

These experiments showed that the capsules reached the specified temperature levels without problems [41]. Some interference problems of the capsule heaters with readings in the control system were observed. These were related to the power supplies of the heaters and were solved by improving their grounding.

#### Capsules Filling with Na

The HFTM capsules are densely packed with EUROFER specimens and it was originally proposed to use the sodium-potassium alloy NaK-78 to fill any void volume to improve the heat conduction and obtain uniform temperature distribution.

**Fig. 25:**
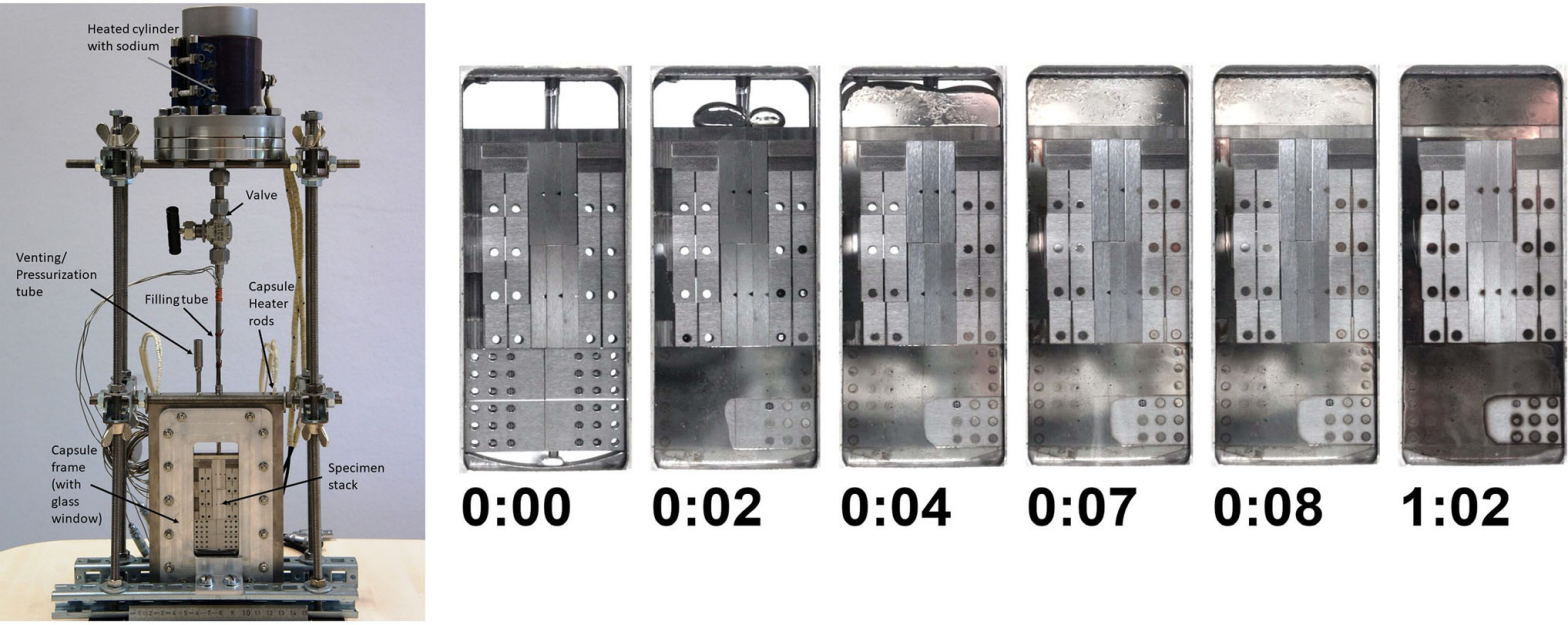
Validation tests on Na filling of the capsules

However, investigations have shown that under neutron irradiation the potassium in the NaK-78 alloy produces argon. This argon generation has the potential to rise massively the internal capsule pressure causing its structural failure. Furthermore, argon gas bubbles can be trapped in the tiny gaps between the specimens in the stack and act as an insulator leading to an inhomogeneous temperature distribution of the specimens. Therefore, it has been decided, in IFMIF-DONES, to replace the NaK-78 with sodium which does not produce argon or other insoluble gases. However, being sodium solid at room temperature and having higher surface tension, filling and wetting processes needed to be demonstrated. Therefore, experiments have been performed at KIT [49] to: (1) test the wettability of EUROFER and 316L stainless steel specimens by sodium and (2) perform a successful sodium filling using a prototype capsule to have a better understanding of how well the specimens are wetted and the gaps are filled with sodium (Fig. 25).

The experiments have shown that at a temperature of 430 °C or higher a good wetting of the EUROFER specimens by sodium is reached. This temperature level is not critical for the filling process. In conclusion, a successful sodium filling of the prototype capsule was demonstrated [41].

#### SSTT Validation

**Fig. 26:**
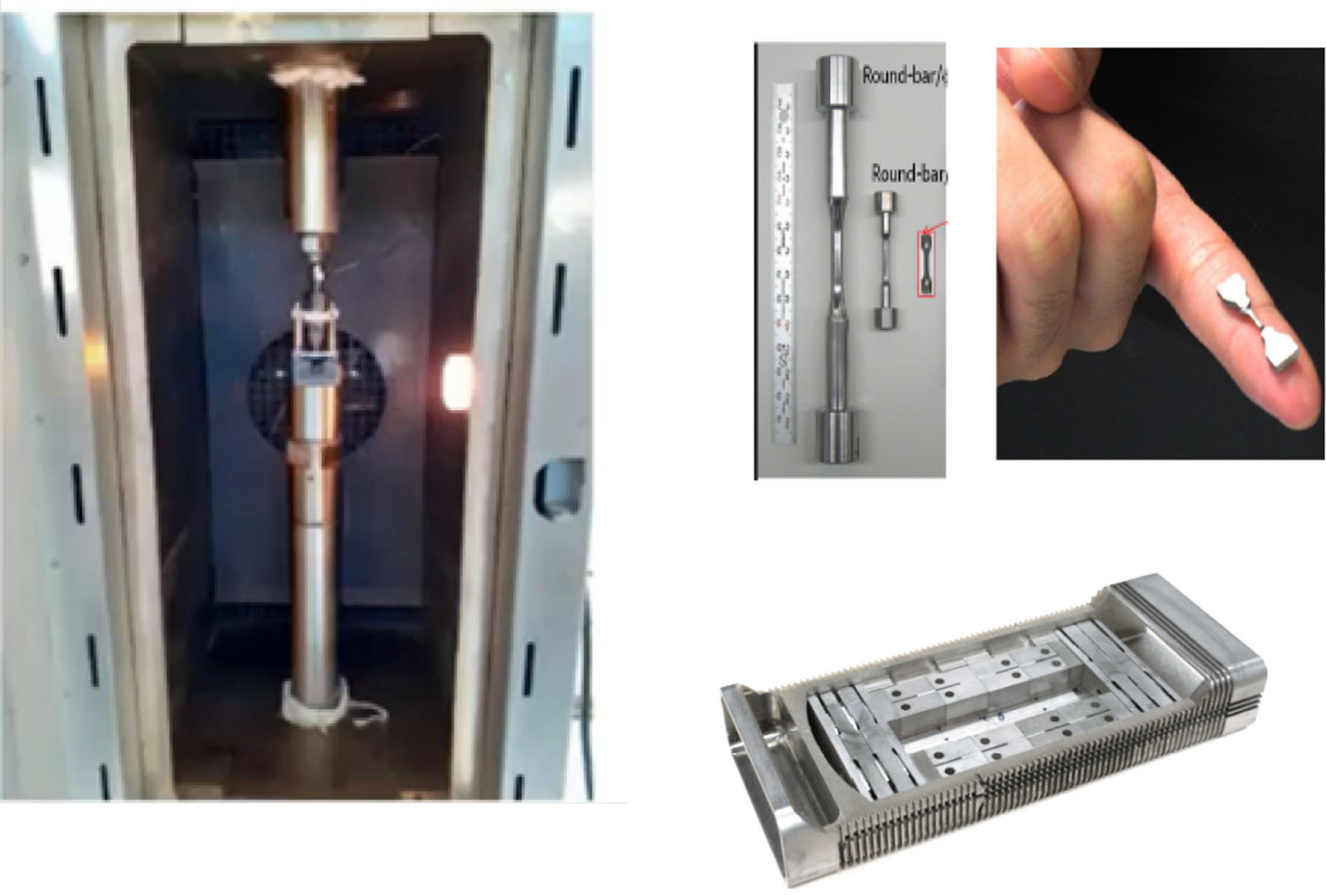
SSTT validation tests

The optimization and standardization of the Small Sample Testing Technique (SSTT) employed for the small size specimens to be irradiated in IFMIF-DONES is a relevant point which needs to be addressed, especially in connection with the licensing process of the future fusion reactors. Thus, validation tasks on sample geometry and testing procedures have been going on at CIEMAT (Spain) as part of the FP8 WPENS work programme. Fabrication and testing of small samples have been performed to confirm their suitability and to optimize the testing procedures (Fig. 26). Although the long experience gained over many years [50] has confirmed that the SSTT methodology is a fully reliable approach, validation efforts in this field still need to be carried out particularly in regard with the optimization and standardization of the tests. Presently, in FP9, most of the SSTT work has been moved to the EUROfusion WPMAT workpackage yet maintaining a link to WPENS for what concerns the specific implementation of SSTT in IFMIF-DONES (e.g., the optimization of the samples arrangement inside the capsule).

### RH Validation

#### RH Validation Tests of TAA Replacement

**Fig. 27:**
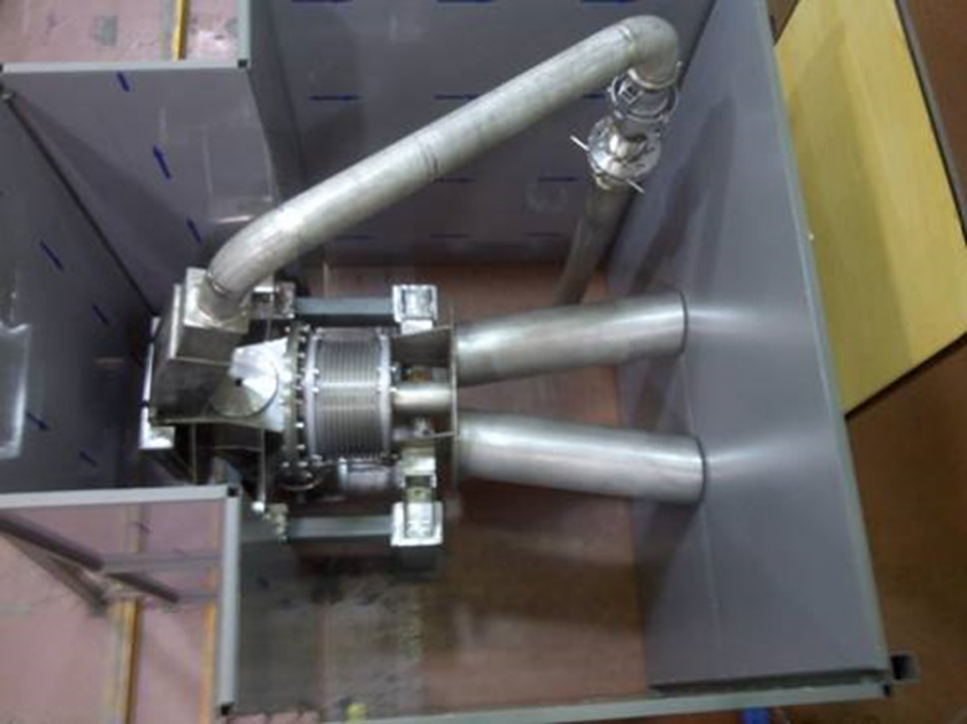
Target System prototype installed at ENEA DRP laboratory

**Fig. 28:**
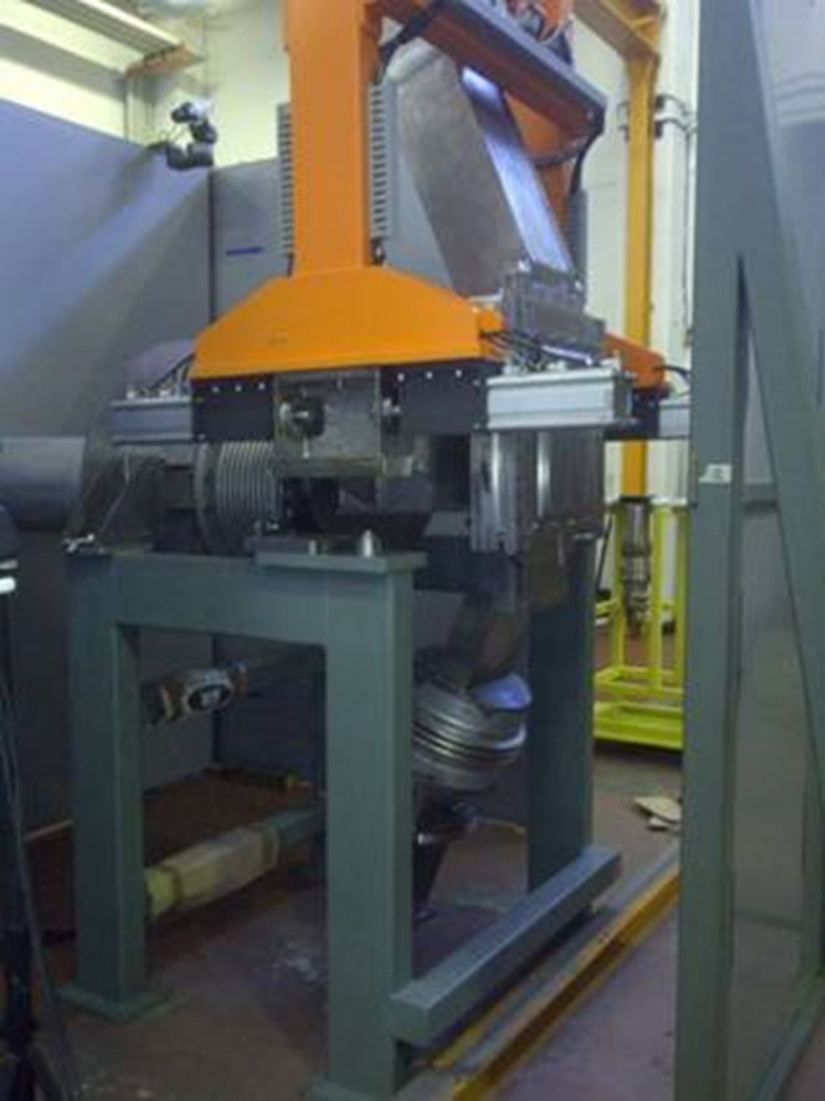
RH validation tests for the replacement of the Target Assembly

Due to the strong irradiation in the TC environment, the Target Assembly is scheduled for annual replacement during the yearly long beam stop period (20 days) of the facility. Therefore, the replacement operations must be carried out inside the test cell by RH devices. The necessary RH procedures and tools were developed and tested by ENEA using the full-scale mockup of the IFMIF target (Fig. 27) during the IFMIF/EVEDA project. In the ENEA Brasimone DRP facility, a dedicated test environment simulating the IFMIF test cell was set up. In this environment, a support for the target assembly was placed and the complete target with attached lithium pipes could be handled. A crane with a suspended mounting plate, customized lift frames for the TAA, a telescopic mast for lifting and lowering the lift frame and a robotic arm holding the bolting tool were used for the operations (Fig. 28).

The RH tests confirmed the feasibility of the planned operations and provided good estimates for the required times (the whole TAA replacement could be done in less than 3 days). Furthermore, a list of detailed improvements to make the TAA design better suited for RH was derived [41].

Nevertheless, it should be pointed out that the TAA design for IFMIF-DONES is slightly different from the one tested, at least regarding the number of connections and how these connections are managed. Also, the layout of the lithium pipes has been changed. Although the suggested improvements in the design of the TAA are still valid, the intervention time for the maintenance of the new TAA design has to be assessed again and maybe further modifications will be required once the validation of the new TAA concept will be completed. This assessment will be part of the RH validation tasks in the FP9 work programme.

## Project Evolution and Achieved Milestones

Since 2015, the development of IFMIF-DONES has constantly progressed within the FP8 EUROfusion WPENS, from the starting conceptual design – based on the original IFMIF concept – to the current baseline design [51–54]. The results of this work have been collected and compiled into a documentation package called the Preliminary Engineering Design Report (PEDR). The first version of the PEDR was issued in 2017. The PEDR contains several documents which include an executive summary, a comprehensive Plant Description Document (PDD), many Technical Annexes to the PDD describing transversal activities (such as, for example, Safety analyses; RAMI studies; Operation and Maintenance activities, 3D CAD models, etc…) and all the Design Description Documents (DDDs) detailing the design of each of the IFMIF-DONES systems. The initial version of the PEDR was updated in 2018 and further updated again in 2019 with site-specific information customized for the Granada site in Spain. During 2020, a special effort was made to produce a final update of the PEDR which was actually issued in 2021 [55] due to the delays caused by the COVID-19 pandemic.

A further important achievement of the project was the compilation of the complete Preliminary Safety Analysis Report (PSAR) which was issued in 2019 and later updated in 2020. This document, which formed the basis for the Safety Analysis Report (SAR) recently released [56], represents a very important step for the IFMIF-DONES construction as it will be used as a reference in the licensing and permitting process of the facility [57].

Another important achievement was the inclusion of IFMIF-DONES, in 2018, in the list of the most strategic European research facilities by the Strategy Forum on Research Infrastructures (ESFRI). This has opened up the possibility to apply for EU funding for the preparation of the project construction activities in the framework of the Horizon 2020 project DONES-PreP (DONES-Preparatory Phase). The main objective of the DONES-PreP project, which ended in December 2021, was to draft the Consortium Agreement for the construction phase of facility in Granada, focusing in particular on the governance, legal and financial aspects.

Finally, an important step of the IFMIF-DONES activities during FP8 took place in 2019 with the organization of three external design reviews (DRw) for selected areas of the project, namely Safety and Neutronics; Logistics and Remote Handling; and Buildings and Plant Systems. The objectives of the DRw were to verify and ensure the adequacy of the analyses and of the design carried out in the concerned areas. The review panels consisted of recognized experts representing international organizations and projects similar to IFMIF-DONES (e.g. CERN, ESS, ITER). As general conclusion of the DRw meetings, the quality of the work developed was rated very high. Nevertheless, the review panels identified some management and design issues which were discussed and summarized in the DRw reports along with a number of proposed corrective actions. The actions were duly implemented in the design work during the following year.

## Conclusions and Future Perspectives

The main achievement of the H2020 (FP8) Early Neutron Source work package is a consolidated preliminary engineering design baseline for the IFMIF-DONES facility. During the course of the project, the earlier conceptual design based on the previous IFMIF configuration has evolved, some aspects have been reviewed and re-designed, some necessary validation activities have been proposed and carried out. Also, important validation results and experience were obtained from the activities performed in the past IFMIF/EVEDA project and in the framework of the Broader Approach collaboration with Japan, which is still going on, and then incorporated in the IFMIF-DONES design baseline.

The present maturity level of the IFMIF-DONES design allowed to define the working programme of the Horizon Europe (FP9) Early Neutron Source work package which will be focused on completing the engineering design of the facility; on performing the remaining experimental validation and qualification activities; on making all the required transversal analyses and on preparing the technical specifications for the launch of the tenders in view of the construction of IFMIF-DONES infrastructure, components and equipment. The present status will allow a smooth transfer of the engineering design of IFMIF-DONES to the project team in charge of the construction of the facility.

## Data Availability

All of the data generated or analysed during this study are included in this published article or can be retrieved from the documents listed in the Reference list. In case, they can also be made available by the corresponding author on reasonable request.

## References

[1] Federici G (2017). European DEMO design strategy and consequences for materials. Nucl. Fus..

[2] Stork D (2014). Materials R&D for a timely DEMO: key findings and recommendations of the EU Roadmap Materials Assessment Group. Fus. Eng. Des..

[3] Stork D (2014). Developing structural, high-heat flux and plasma facing materials for a near-term DEMO fusion power plant: the EU assessment. J. Nucl. Mater..

[4] Stork D (2017). Towards a programme of testing and qualification for structural and plasma-facing materials in fusion neutron environments. Nucl. Fus..

[5] Hagiwara M (2005). Measurement of Neutron Emission Spectra in Li(d, xn) Reaction with thick and thin targets for 40 MeV deuterons. Fus. Sci. Technol..

[6] Knaster J (2018). An assessment of the available alternatives for fusion relevant neutron sources. Nucl. Fusion.

[7] Goland N (1975). Use of Li(d, n) neutrons for simulation of radiation effects in fusion reactors. IEEE Trans. Nucl. Sci..

[8] Grand P (1976). An intense Li(d, n) neutron radiation test facility for controlled thermonuclear reactor materials testing. Nucl. Technol..

[9] Pottmeyer EW (1979). The fusion materials irradiation test facility at Hanford. J. Nucl. Mater..

[10] K. Ehrlich, E. Daum (eds), *Proceedings of IEA Workshop on Selection of Intense Neutron Sources* (Karlsruhe, Germany, September 1992) KfK Report 5296 (May 1994). https://publikationen.bibliothek.kit.edu/270033342/3813467

[11] M. Martone (ed), *IFMIF—Conceptual Design Activity Final Report IFMIF-CDA-Team ENEA-RT/ERG/FUS/9611-Report *(1996). https://www.osti.gov/etdeweb/biblio/479522

[12] IFMIF International Team, *IFMIF Comprehensive Design Report*, 2004, upon request to ifmif@ifmif.org

[13] Knaster J (2015). The accomplishment of the engineering design activities of IFMIF/EVEDA: The European – Japanese project towards a Li(d, xn) fusion relevant neutron source”. Nucl. Fus..

[14] Perez M (2015). The engineering design evolution of IFMIF: from CDR to EDA Phase. Fus. Eng. Des..

[15] IFMIF/EVEDA Integrated Project Team, *IFMIF Intermediate Engineering Design Report Plant (IIEDR)*, 2013, upon request to ifmif@ifmif.org

[16] T. Donné et al., *European Research Roadmap to the Realisation of Fusion Energy*. EUROfusion Consortium (2018). http://www.euro-fusion.org/eurofusion/roadmap

[17] Ibarra A (2014). A stepped approach from IFMIF/EVEDA toward IFMIF. Fus. Sci. Technol..

[18] Arbeiter F (2018). Planned material irradiation capabilities of IFMIF-DONES. Nuclear Mater. Energy.

[19] Qiu Y, Arbeiter F, Fischer U, Schwab F (2018). IFMIF-DONES HFTM neutronics modeling and nuclear response analyses. Nuclear Mater. Energy.

[20] J. Henry, L. Ciupinski, D. Jimenez Rey, M. R. O’Brien, H.-C. Schneider, M. Serrano, D. Terentyev, Evaluation of facilities that could be used for the characterization of the samples irradiated in the DONES facility, DONES-PreP Project (No.870186) Deliverable D9.2–2 (2021)

[21] I. Podadera et al., The accelerator system of IFMIF-DONES multi-MW facility, in *Proceedings of 12th International Particle Accelerator Conference (IPAC'21)*, Campinas, Brazil, May 2021, pp. 1910–1913. 10.18429/JACoW-IPAC2021-TUPAB211

[22] Gobin R (2016). Installation and first operation of the International Fusion Materials Irradiation Facility injector at the Rokkasho site. Rev. Sci. Instrum..

[23] A. Pisent et al., IFMIF-EVEDA RFQ design, in *Proceedings of 11th European Particle Accelerator Conference (EPAC’08)*, Genova, Italy, June 2008, paper THPP078, pp. 3542–3544. https://accelconf.web.cern.ch/e08/papers/thpp078.pdf

[24] F. Scantamburlo et al., LIPAC, the IFMIF/EVEDA prototye accelerator: alignment and assembly current status and possible future improvements, in *14th International Workshop on Accelerator Alignment*, ESRF, Grenoble, France, October 3–7, 2016. https://www.slac.stanford.edu/econf/C1610034/papers/832.pdf

[25] I. Podadera et al., Manufacturing, assembly and tests of the LIPAc medium energy beam transport line (MEBT), in *Proceedings of 28th Linear Accelerator Conference (LINAC’16)*, East Lansing, MI, USA, September 2016, pp. 554–557. 10.18429/JACoW-LINAC2016-TUPLR041

[26] T. Plomion et al., Preliminary design of the IFMIF-DONES superconducting linac, in *Proceeding of 19th International Conference RF Superconductivity (SRF’19)*, Dresden, Germany, June-July 2019, pp. 311–314. 10.18429/JACoW-SRF2019-MOP097

[27] Regidor D (2021). IFMIF-DONES RF System. Fus. Eng. Design.

[28] Nomen O (2020). Preliminary design of HEBT of IFMIF-DONES. Fus. Eng. Design.

[29] F. Grespan et al., IFMIF/EVEDA RFQ beam commissioning at nominal 125 mA Deuteron beam in pulsed mode, *Presented at the 11th International Particle Accelerator Conference (IPAC’20)*, Caen, France, May 2020, paper TUVIR11. 10.18429/JACoW-IPAC2020-TUVIR11

[30] de la Morena C (2021). First validation experiments of the prototype solid state RF system for IFMIF-DONES. Fus. Eng. Des..

[31] Dézsi T, Nitti FS, Tóth M, Pásti Sz, Balogh B, Ibarra A (2019). Overview of the current status of IFMIF-DONES secondary heat removal system design. Fus. Eng. Des..

[32] Arena P (2019). The design of the DONES lithium target system. Fus. Eng. Des..

[33] Simon S, Dézsi T, Arbeiter F, Tóth M, Castellanos J, Ibarra A (2021). Thermal-hydraulic simulation of IFMIF-DONES Test Cell atmosphere. Fus. Eng. Des..

[34] Tian K (2019). Preliminary analysis on a Maintainable Test Cell concept for IFMIF-DONES. Fus. Eng. Des..

[35] Katona I (2022). Preliminary finite element analysis of the stainless-steel liner of the maintainable test cell concept of IFMIF-DONES. Nucl. Mater. Energy.

[36] Wiącek U (2021). New approach to the conceptual design of STUMM: a module dedicated to the monitoring of neutron and gamma radiation fields generated in IFMIF-DONES. Fus. Eng. Des..

[37] A. Maj, M.N. Harakeh, M. Lewitowicz, A. Ibarra, W. Królas, *White Book on the Complementary Scientific Program at IFMIF-DONES, IFJ PAN Report 2094/PL, 2016*. https://rifj.ifj.edu.pl/handle/item/78

[38] Cappelli M, Centioli C, Neri C, Monti C, Ibarra A (2019). IFMIF-DONES Central instrumentation and control systems: general overview. Fus. Eng. Des..

[39] T. Tadic, *Final report on the Decommissioning Plan, IDM ref. EFDA_D_2NYYTS* (2021), upon request to the corresponding author

[40] Arbeiter F (2018). The accomplishments of lithium target and test facility validation activities in the IFMIF/EVEDA phase. Nucl. Fus..

[41] B. Brañas, *Consequences of Validation Activities on IFMIF-DONES Engineering Design*, upon request to the corresponding author

[42] Dzitko H (2021). Status and future development of the LIPAc. Fus. Eng. Des..

[43] Kondo H (2012). Completion of IFMIF/EVEDA Li test loop construction. Fus. Eng. Des..

[44] Knaster J (2017). Overview of the IFMIF/EVEDA project. Nucl. Fus..

[45] Aiello A (2013). Lifus (Li for Fusion) 6 loop design and construction. Fus. Eng. Des..

[46] Arbeiter F (2016). Design description and validation results for the IFMIF High Flux Test Module as outcome of the EVEDA phase. Nucl. Mater. Energy.

[47] Vladimirov P, Möslang A, Marmy P (2008). Nuclear responses in IFMIF creep-fatigue testing machine. Fus. Eng. Des..

[48] Knaster J, Favuzza P (2017). Assessment of corrosion phenomena in liquid lithium at T < 873 K. A Li(d, n) neutron source as case study. Fus. Eng. Des..

[49] Abou-Sena Ali, Arbeiter Frederik, Schwab Florian, Zinn Kevin (2019). Using sodium as filling and heat-conducting material in the irradiation capsules of the fusion materials irradiation facility IFMIF-DONES. Fusion Engineering and Design.

[50] Lucas GE (1983). The development of small specimen mechanical test techniques. J. Nucl. Mater..

[51] Ibarra A (2018). The IFMIF-DONES project: preliminary engineering design. Nucl. Fus..

[52] Ibarra A (2019). The European approach to the fusion-like neutron source: the IFMIF-DONES project. Nucl. Fus..

[53] Bernardi D (2019). Towards the EU fusion-oriented neutron source: the Preliminary Engineering Design of IFMIF-DONES. Fus. Eng. Des..

[54] Królas W (2021). The IFMIF-DONES fusion oriented neutron source: evolution of the design. Nucl. Fus..

[55] IFMIF-DONES team, *Preliminary Engineering Design Report (PEDR) v3.0, October 2021*, upon request to the corresponding author

[56] IFMIF-DONES Safety Team, *Safety Analysis Report (SAR) v1.0, November 2021*, upon request to the corresponding author

[57] Martín-Fuertes F (2019). Integration of safety in IFMIF-DONES design. Safety.

